# Stereoselective Biotransformation: Transfer of Learning to Advance Drug Metabolism and Biocatalysis

**DOI:** 10.1002/anie.202526152

**Published:** 2026-04-24

**Authors:** Grace A. Okunlola, Godwin A. Aleku

**Affiliations:** ^1^ Institute of Pharmaceutical Science King's College London London UK

**Keywords:** biocatalysis, biotransformation, drug metabolism, drug metabolizing enzymes, stereoselectivity

## Abstract

Chirality is an important determinant of drug action, as enantiomers can exhibit markedly different pharmacological and toxicological profiles. Although the importance of stereochemistry in drug efficacy is well established, its role in drug metabolism and disposition remains comparatively underexplored, despite the inherently stereoselective nature of drug metabolizing enzymes. Given the high prevalence of chiral drugs in clinical use and among newly approved drugs, a systematic evaluation of stereoselective drug metabolism is needed. Understanding stereoselective biotransformations has important implications for predicting drug disposition and response and may also inspire novel biocatalytic and biomimetic strategies to address challenges in enantioselective synthesis of chiral active pharmaceutical ingredients and their metabolites. In this Systematic Review, we examine current trends and practices in the investigation of stereoselectivity in drug metabolism, the key factors influencing stereoselective metabolism, and the associated challenges and opportunities. We highlight how biocatalytic approaches can improve stereoselective access to chiral metabolites, and how insights from drug metabolism and pharmacokinetics (DMPK) studies can inspire the development of novel biocatalytic and biomimetic synthesis routes. Transfer of learning and cross‑disciplinary collaboration between biocatalysis and DMPK scientists will be critical for accelerating progress in these areas and for addressing shared challenges, including stereoselectivity prediction.

## Graphical abstract

Understanding stereoselective biotransformations has implications for predicting drug disposition and response and may also inspire novel biocatalytic and biomimetic strategies to address challenges in metabolite and API synthesis.

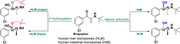

**Abbreviations**
11βHSD: 11β‐hydroxysteroid dehydrogenase
2APCoAE/2APCoE: 2‐arylpropionyl‐coenzima A epimerase
ADH: alcohol dehydrogenase
ADME: absorption, distribution, metabolism, and excretion
AKR: aldoketo reductases
ALDH: aldehyde dehydrogenase
API: active pharmaceutical ingredient (API synthesis)
BChE: butyrylcholinesterase
BLQ: below the limit of quantification
BVO: Baeyer–Villiger oxidation
CES: carboxylesterase
CIP: Cahn–Ingold–Prelog
COMT: catechol–O–methyltransferase
CYP450 / CYP: cytochrome P450 monooxygenase
DDI: drug–drug interaction
DME: drug metabolizing enzyme
DMPK: drug metabolism and pharmacokinetics
DMS: drug metabolism studies
GLYAT: glycine *N*‐acetyltransferase
GST: glutathione S‐transferase
HIM: human intestine microsomes
HLC: human liver cytosols
HLM: human liver microsomes
HLS9: human liver S9 fractions
MAO: monoamine oxidase
MDMA: 3,4‐methylenedioxymethamphetamine
MEH: microsomal epoxide hydrolase
NAD(P)(H): nicotinamide cofactors (oxidized and reduced forms)
NAT: N‐acetyltransferase
PAPS: 3′‐phosphoadenosine‐5′‐phosphosulfate
PBPK: physiologically based pharmacokinetic model
PK/PD: pharmacokinetics/pharmacodynamics
SAM: S‐adenosylmethionine
SDM: stereoselective drug metabolism
SULT: sulfotransferase
UDPGA: uridine 5′‐diphosphoglucuronic acid
UGT: UDP‐glucuronosyltransferase
UPO: unspecific peroxygenase

## 1 Introduction

Chiral molecules occur as distinct enantiomers (*R‐* vs. *S*‐configurations, “right‐handed” vs. “left‐handed” configurations) that are chemically identical, only differing in the spatial arrangement of substituents attached to the stereogenic center. The distinct configurations of individual enantiomers can confer unique modes of interaction with a drug target. As a result, an enantiomeric pair of a drug can elicit profoundly different pharmacological and toxicological profiles, sometimes with clinically significant implications if the racemic mixture is administered. Often, one of the enantiomeric pair (the eutomer) exhibits the desired biological activity, while the other (the distomer) may display only weak activity, lack the desired activity, or elicit undesirable biological effects.

Medicinal compounds with one or multiple chiral centers, often referred to as chiral drugs, constitute a significant proportion of medicines used in the clinic. Chiral drugs were predominantly marketed as racemic mixtures, but a 1992 landmark regulation brought stricter regulatory requirements [1], resulting in a notable shift toward the registration of single enantiomer drugs [2]. However, considering historical approvals, a significant proportion of chiral drugs used in clinics are racemates [3]. It is also possible, albeit rarely, that enantiomers can exhibit comparable biological activity, and there have been reported cases where the administration of a racemic mixture has led to an overall beneficial therapeutic outcome compared to the single enantiomer formulation [4]. Thus, significantly fewer racemic drugs are currently being approved [2].

The impact of chirality extends beyond the differential biological action of chiral drugs; it plays a key role in drug metabolism and disposition, with implications on the overall therapeutic outcome of pharmacological treatments [5, 6, 7]. A notable example, both from pharmacodynamic and drug metabolism viewpoints, is the tragic thalidomide. Racemic thalidomide was used as an antiemetic in the 1950s–1960s until its (*S*)‐enantiomer was linked to severe congenital disabilities. Attempts to use enantiopure (*R*)‐thalidomide failed due to its in vivo chiral inversion to the teratogenic (*S*)‐form [8]. Chiral inversion has become a recognized phenomenon in drug metabolism, with a significant proportion of these inversions mediated by drug metabolizing enzymes (DMEs) [9, 10].

**SCHEME 1:**
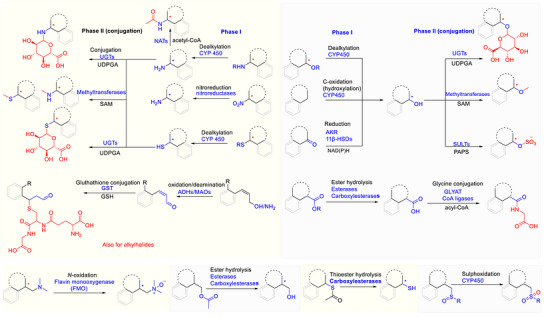
An overview of biotransformation reactions in drug metabolism and the associated drug metabolizing enzymes. Cofactors: PAPS – 3′‑phosphoadenosine‑5′‑phosphosulfate; UDPGA – uridine 5′‑diphospho‑glucuronic acid; SAM – S‑adenosyl‑methionine; NAD(P)(H) – nicotinamide cofactors. AKRs, aldo‐keto reductases; CYP450, cytochrome P450 monooxygenases; GLYATs, glycine *N*‐acetyltransferases; GSTs, glutathione S‐transferases; MAOs, monoamine oxidases; NATs, *N*‐acetyltransferases; SULTs, sulfotransferases; UGTs, UDP‐glucuronosyltransferases; 11β‐HSD, 11β‐hydroxysteroid dehydrogenase.

Broadly, chemical modifications to drugs by drug metabolizing enzymes (DMEs) are essential bioactivation or detoxification mechanisms within key organs (e.g., liver) in biological systems. In humans and other non‐human primates, these biotransformation reactions are broadly categorized as Phase I and Phase II reactions. Functionalization reactions, mainly through oxidation, reduction, and hydrolysis, catalyzed by a broad range of oxidoreductases, hydrolases, and lyases, are categorized as Phase I reactions. These reactions generate nucleophilic groups (e.g., ─NH_2_, ─SH, ─OH) or electrophilic groups (e.g., ─CHO, ─CO─R, ─COOH, R─SO_2_─R′) which are then conjugated to endogenously sourced polar moieties such as glucuronic acid (glucuronidation), sulfate (sulfation), acetyl (acetylation), glutathione (glutathione conjugation), amino acids (amino acid conjugation), and methyl groups (methylation), Scheme 1. These Phase II modifications are catalyzed predominantly by transferases, including UDP‐glucuronosyltransferases (UGTs), sulfotransferases (SULTs), glutathione S‐transferases (GSTs), *N*‐acetyltransferases (NATs)/glycine *N*‐acetyltransferases (GLYATs), and methyltransferases (Scheme 1). The resulting metabolites from these transformations are usually significantly more polar than their parent drugs, making them readily excretable mainly in urine. Several recent systematic analyses of the metabolism of commonly prescribed and newly approved drugs showed consistently that cytochrome P450 monooxygenases (CYP450), UGTs, esterases, flavin‐containing monooxygenases (FMOs), *N*‐acetyltransferases (NATs), and monoamine oxidases (MAOs) are the major DMEs, with CYPs and UGTs catalyzing the majority of Phase I and Phase II reactions, respectively [11, 12, 13].

In vitro, in vivo *(in animal models)*, and ex vivo drug metabolism studies are integral to preclinical drug development projects. When appropriate models are carefully selected, these studies, often performed in combination, provide crucial and reliable information about the metabolic fate of compounds, and tend to correlate with observations in human in vivo clinical studies [14, 15]. An AstraZeneca retrospective comparative analysis of metabolite profiles obtained from in vitro and in vivo preclinical studies for 27 of their drug candidates found that 81% of human circulating metabolites of these drugs were previously seen in preclinical in vitro and/or in vivo experiments [16]. Nevertheless, metabolism studies are extended to clinical development, as the identification of new major metabolites not observed in preclinical animal studies or known metabolites occurring at significantly higher concentrations in humans than observed in animal studies (disproportionate metabolites) remains a possibility [14, 17, 18, 19, 20]. Major regulatory agencies have developed frameworks and guidelines on drug metabolism studies in clinical trials. For example, the FDA has detailed guidance on metabolites in safety testing (MIST), which, among other requirements, stipulates that metabolites that occur at a level greater than 10% of total drug‐related exposure in humans and are not observed during animal testing must undergo further thorough preclinical investigations [21].

A long‐standing problem with pharmaceutical treatments is the heterogeneity in drug responses across populations and individuals [22, 23]. An established and significant contributor to this heterogeneity is differences in drug metabolism rates, driven by genetic variations in drug metabolizing enzymes (genetic polymorphisms) [24, 25], drug‐drug interactions (DDIs), and other biological factors. In the clinic, comorbidities are common, and treatment regimens involve the concomitant administration of two or more drugs (polypharmacy). The metabolism of one drug may be altered by the other if the two co‐administered drugs are both substrates of the same DME [26]. For example, the first‐line regimen for treating tuberculosis involves administering isoniazid and rifampicin, along with other antitubercular agents, for several months. The primary metabolic route for isoniazid *N*‐acetylation is catalyzed by *N*‐acetyltransferase 2 (NAT2), and patients with low‐activity alleles of NAT2, as well as fast acetylators, are at a significantly increased risk of serious side effects or therapeutic failure [27]. On the other hand, rifampicin is a known potent inducer of CYP3A4 and can also induce UGT1A1 [28]. These two enzymes are involved in the metabolism of several other drugs and clinically significant drug‐drug interactions (DDIs) can occur when substrates of these enzymes are concomitantly administered with rifampicin.

There is growing evidence that the stereoselectivity in the metabolism of chiral and prochiral drugs plays a vital role in their differential metabolism. However, current literature has focused on the influence of chirality in toxicology and pharmacodynamic properties [3, 29, 30, 31]. Research efforts on stereoselectivity in drug metabolism are fragmented. The synthesis and assessment of progress in this area in readily accessible Reviews are scant, with the last related Reviews published in 2007 and 2009 [5, 32]. Other relatively recent Reviews have covered analytical methods employed in assessing stereoselectivity in drug metabolism [30, 32] or have focused on general concepts and definitions on the nature of stereoselective biotransformation (e.g., substrate vs. product stereoselectivity) [33]. The lack of more recent critical reviews on stereoselectivity in metabolism has hampered a clearer understanding of the factors that govern stereoselective drug metabolism (SDM), their potential impact on treatment outcomes, and interventions to minimize the variability introduced by stereoselective biotransformations. Stereoselectivity in drug metabolism warrants closer evaluation, given the proportion of chiral drugs in clinical use and in new drug approvals [2, 34], as well as the inherent stereoselective nature of DMEs. An assessment of the various stereoselective biotransformation reactions in drug metabolism can also inspire the development of novel biocatalytic and biomimetic synthesis approaches to address current gaps in metabolite synthesis and sustainable pharmaceutical synthesis.

To address these points and identify trends and gaps in the assessment of stereoselectivity in drug metabolism, we conducted a Systematic Review. A comprehensive search was performed across four major databases: Embase, Medline, PubMed, and Web of Science—to retrieve studies published between January 2014 and February 2025. Following the exclusion of ineligible studies according to the criteria outlined in the PRISMA flow diagram (Figure S1, Table S1), a total of 43 studies were analyzed. Here, we discuss trends and practices in stereoselectivity investigations, factors influencing stereoselective metabolism, and the associated challenges and opportunities in this field.

## 2 Trends in the Investigations of Stereoselectivity in Drug Metabolism

**FIGURE 1:**
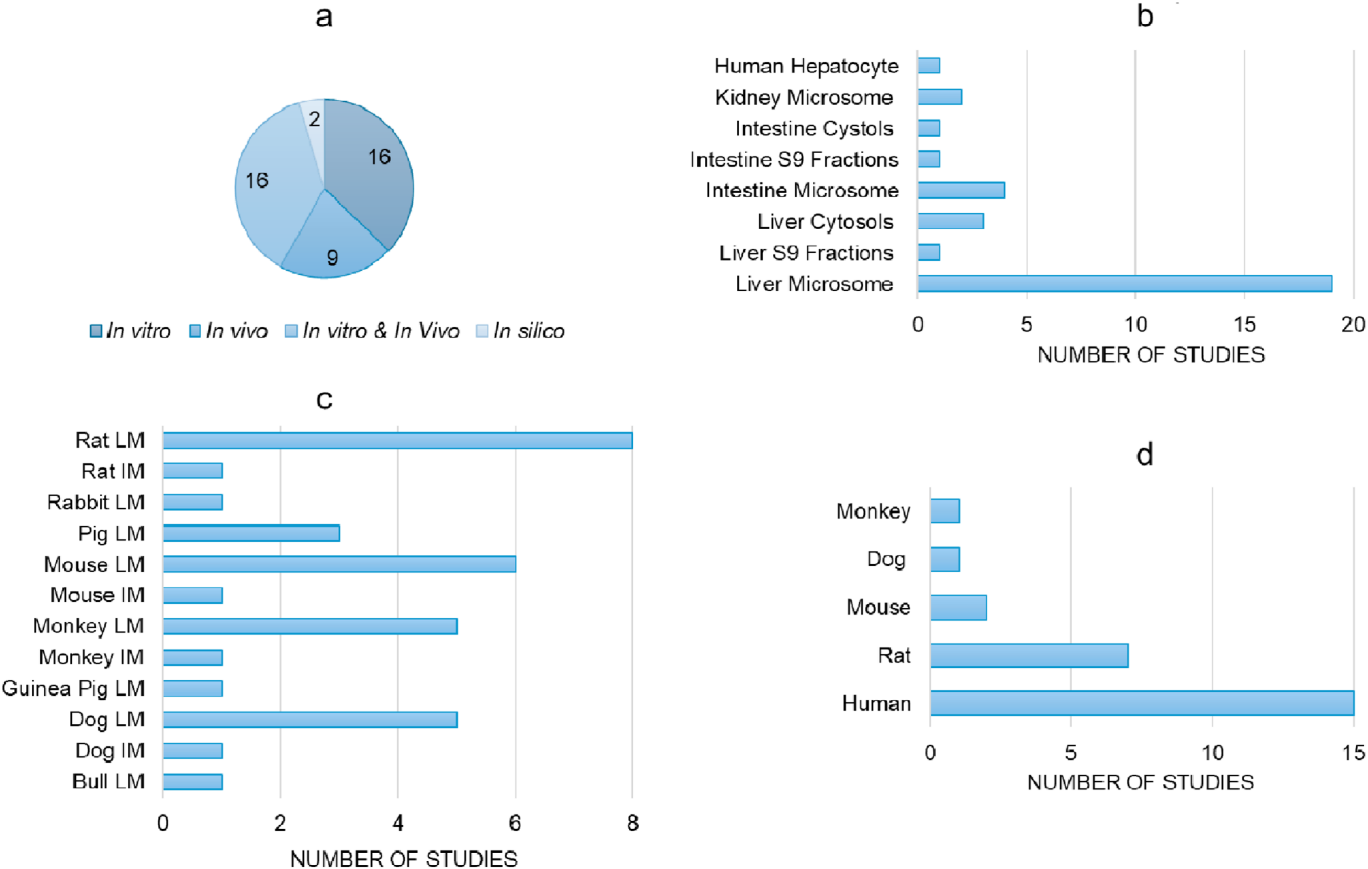
Types of models used for drug metabolism studies. (a) In vitro, in vivo, and in silico studies reporting stereoselective drug metabolism. (b) Human subcellular fractions frequently used as in vitro models of drug metabolism studies. (c) Non‐human organ fractions frequently used as in vitro models of drug metabolism studies. (d) Animals frequently used as in vivo models for drug metabolism studies. IM, intestinal microsomes; LM, liver microsomes.

We analyzed the 43 eligible studies retrieved from our systematic search to identify the techniques, models, and (sub)cellular preparations most frequently employed to assess stereoselectivity in drug metabolism. Seventy‐four percent of the studies (*n* = 32) used in vitro techniques, either alone (*n* = 16) or supplemented with in vivo applications (*n* = 16) (Figure 1a). The most frequently used in vitro approach for investigating stereoselectivity was incubating racemic and individual enantiomeric forms of a chiral drug with subcellular fractions and/or isolated recombinant enzymes.

Biotransformation reactions to assess stereoselectivity were frequently performed with human liver microsomes (HLMs), because microsomes from the main metabolism organ, the liver, are rich in membrane‐bound CYPs and UGTs. Other subcellular preparations, which contain only soluble enzymes such as human liver cytosols (HLCs) and human liver S9 fractions, were also used, albeit less frequently. Stereoselective drug metabolism (SDM) was reported for 8 non‐human hepatic microsomes, with microsomes from rat, mouse, monkey, and dog being the most employed subcellular preparations for in vitro studies (Figure 1c). Similarly, rats, mice, monkeys, and dogs were also used as non‐human in vivo models for SDM and drug clearance studies (Figure 1d). While some of the data obtained with these species were consistent with those observed in the equivalent human models, interspecies differences in stereoselectivity were observed across multiple drugs.

Given our focus on stereoselective biotransformation, our systematic literature searches identified only four studies that used primary hepatocytes to investigate stereoselective metabolism. However, only one of these studies, conducted by Walker and colleagues [35], met our criteria for detailed analysis. They examined the biotransformation of ^13^C‐labeled primaquine (PQ) and its two enantiomers, (+)‐PQ and (−)‐PQ, using pooled mixed‐gender cryopreserved primary human hepatocytes. Two major Phase I pathways, oxidative deamination and hydroxylation, were both stereoselective. Initial rate measurements showed that oxidative deamination of the primary amine group of PQ generated the corresponding aldehyde at 8 times higher levels when (−)‐PQ was the substrate than with (+)‐PQ, whereas a major quinoline oxidation product was formed preferentially from (+)‐PQ. Similarly, PQ Phase II conjugation reactions were also stereoselective. While the glucuronide conjugates were generated preferentially from (+)‐PQ, the glycosylated and carbamoyl glucuronide conjugates were formed mainly or exclusively from (+)‐PQ, respectively [35]. The other three studies identified in our systematic literature searches investigated stereoselective biotransformation in rat primary hepatocytes but focused on fungicides such as myclobutanil [36], metalaxyl [37], and lactofen [38] and, as such, were not included in our detailed analysis. Earlier work by Koppele and colleagues in the late 1980s and early 1990s also reported stereoselective glutathione conjugation and amidase‐catalyzed hydrolysis in isolated rat hepatocytes [39, 40]. Within the broader context of drug metabolism studies, primary human hepatocytes are considered the gold standard in vitro liver models in preclinical studies. However, in most applications of these models, stereochemical aspects of hepatocyte‐mediated biotransformation reactions were not examined.

Two studies employed in silico techniques such as quantum mechanics/molecular mechanics (QM/MM) [28, 41] or physiologically‐based pharmacokinetic (PBPK) models [42] (Figure 1a). Several computational tools have been developed to predict drug metabolic routes, and an integrated computational and experimental approach for drug metabolism studies has been advocated [43, 44, 45, 46, 47]. However, computational predictions of stereoselectivity remain less reliable, as subtle changes in the energy profiles of intermediates can lead to substantial differences in experimentally observed stereoselectivity [48, 49].

Overall, investigations into stereoselectivity using in vitro approaches have enabled the elucidation of the stereoselective properties of enzymes, as well as the assessment of metabolic kinetics, enzyme inhibition/induction, and individual enantiomer interactions with other drugs. However, subcellular fractions from extrahepatic metabolic sites have not been adequately investigated, as evidenced by the low frequency of intestinal or kidney microsomal fractions used as in vitro models (*n* = 4 and *n* = 2, respectively; Figure 1b). Similarly, stereoselectivity investigations using primary human hepatocytes have remained limited, despite the extensive use of these cells in broader drug metabolism studies. The vital role of extrahepatic organs and tissues, including the gut microbiome, in SDM investigations should therefore no longer be overlooked [50, 51].

More recently, humanized mice models have been increasingly employed to study drug metabolism. Given their relatively recent development, our systematic searches did not identify studies that specifically applied these models to stereoselective metabolism. However, their utility is rapidly expanding and is likely to extend to investigations of stereoselectivity. Several humanized chimeric mouse models have been developed and shown to exhibit human‐like drug metabolism profiles [52, 53, 54, 55], and examples of these models are discussed further in Section 5 of this Review.

## 3 Biotransformation Reactions and the Associated Drug Metabolizing Enzymes

**FIGURE 2:**
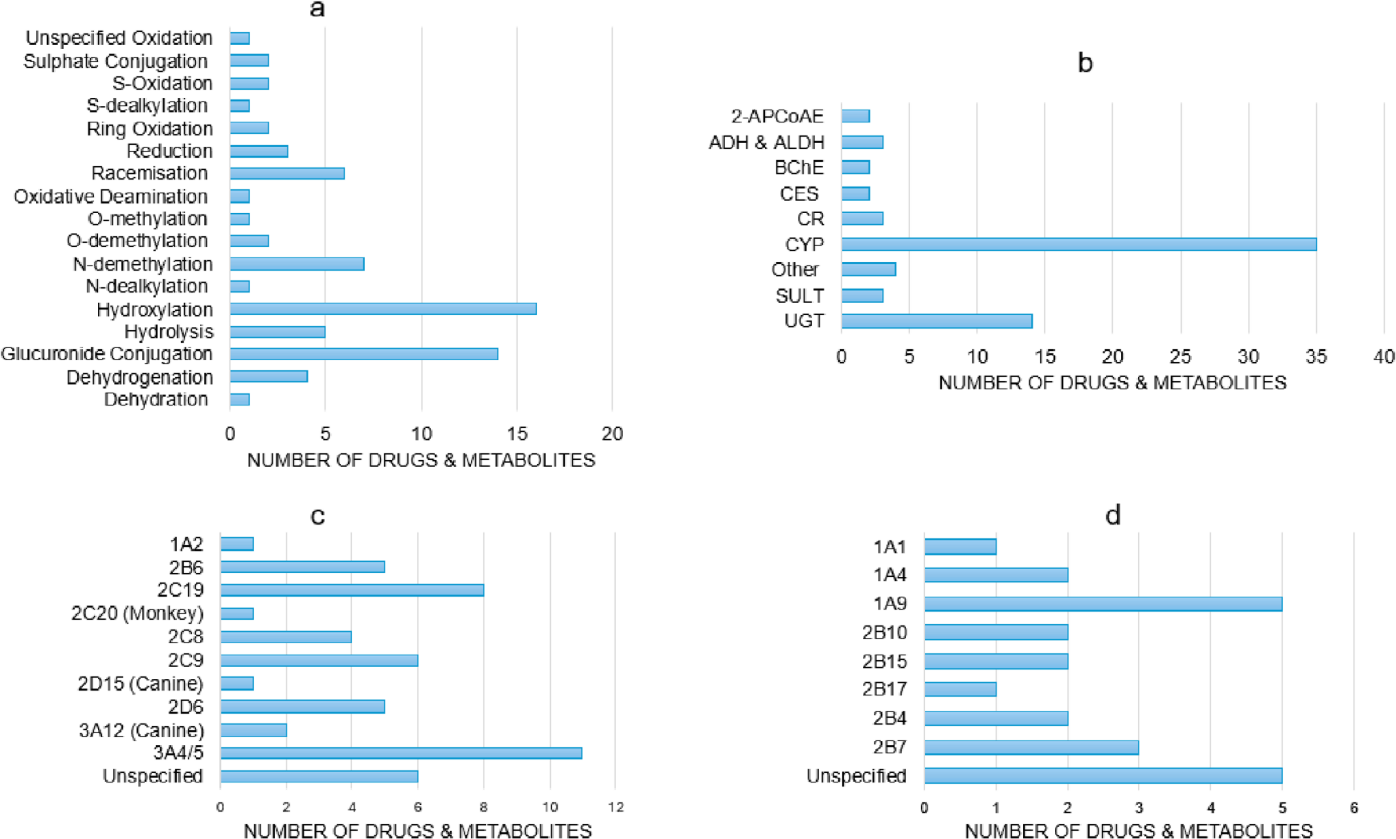
Distribution of reported stereoselective biotransformation reactions and the responsible drug metabolizing enzymes (DMEs). (a) Stereoselective biotransformation frequently reported. (b) DMEs frequently associated with stereoselective drug metabolism (SDM). (c) Frequency of CYP isoforms used in SDM studies. (d) Frequency of UGT isoforms associated with SDM. 2‐APCoE, 2‐aryl propionyl‐coenzyme A epimerase; ADH, alcohol dehydrogenase; ALDH, aldehyde dehydrogenase; BChE, butyrylcholinesterase; CES, carboxylesterase; CR, carbonyl reductases; CYP, cytochrome P450; SULT, sulfotransferase; UGT, UDP‐glucuronosyltransferase.

Our analysis revealed 17 distinct stereoselective biotransformations of 51 chiral compounds, with stereoselective hydroxylation, glucuronide conjugation, and *N*‐demethylation being the most prevalent transformations (Figure 2a). CYPs and UGTs were the most reported enzymes for these stereoselective biotransformations (Figure 2b), consistent with the dominant roles of these enzymes in drug metabolism [11, 13, 56]. Other DMEs catalyzing stereoselective metabolism include sulfotransferases (SULTs); carbonyl reductases such as 11β‐hydroxysteroid dehydrogenase (11β‐HSD) and aldo‐keto reductases (AKR); aldehyde dehydrogenase; alcohol dehydrogenase; 2‐arylpropionyl coenzyme A epimerase; butyrylcholinesterase; carboxylesterase; catechol‐O‐methyltransferase; microsomal epoxide hydrolase; and monoamine oxidase A (Figure 2b).

**SCHEME 2:**
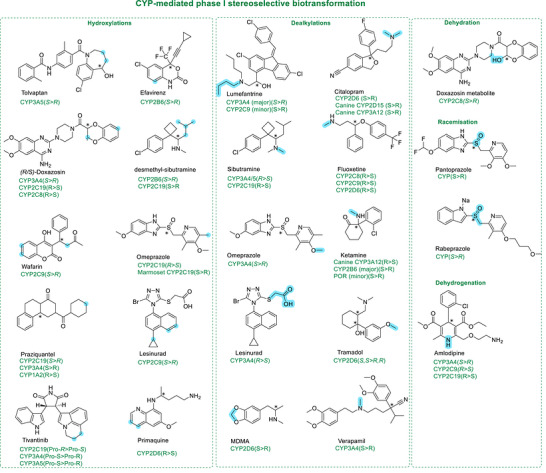
Cytochrome P450 (CYP)‐catalyzed Phase I biotransformation reactions. Sites of reactions are indicated as color highlights. Stereopreference is indicated by the greater‐than symbol (>): for example, S > *R* means the (*S*)‐enantiomer is metabolized faster than the (*R*)‐enantiomer. HMMA, 4‐hydroxy‐3‐methoxymethamphetamine; MDMA, 3,4‐methylenedioxymethamphetamine. See also Tables S2–S5.

Again, consistent with the prevalence of isoforms in drug metabolism [11, 13, 56], CYP3A4/5, 2C19, 2C9, 2B6, and 2D6 (Figure 2c) were the most frequently reported CYP isoforms for SDM, while the most common UGT isoforms associated with stereoselective glucuronidation include 1A9, 2B7, 1A4, 2B10, 2B15, and 2B4 (Figure 2d). When stereoselectivity was assessed using orthologs of human DMEs in commonly used animal models, some correlations and differences in stereoselectivity patterns were observed. Martignoni et al. proposed equivalences between canine CYP2D15 and human CYP2D6, canine CYP3A12 and human CYP3A4/5, and monkey CYP2C20 and human CYP2C8 [57]. In most of these transformations, the enantiomers were metabolized to the corresponding metabolites at different rates, even when the chiral center was remote from the site of reaction (Scheme 2).

CYP enzymes catalyze a broad range of transformations. CYP3A4/5, CYP2C19, CYP2D6, CYP2C8, CYP2C9, and CYP1A2 catalyze the stereoselective hydroxylation of unactivated C−H bonds or aromatic hydroxylation across a broad range of substrates, for example, tolvaptan [58], doxazosin [59], sibutramine [60], omeprazole [41], tucatinib [61], warfarin [62], praziquantel [63], ibuprofen [64], lesinurad [65], tivantinib [66], yielding the corresponding hydroxylated metabolites, with CYP2D6 and CYP2C9 particularly associated with aromatic hydroxylation. The enantiopreference in the rate of hydroxylation appears to be substrate dependent, although the rate of CYP3A4/5‐catalyzed hydroxylation of the (*S*)‐enantiomers was greater than that of the (*R*)‐enantiomers for most substrates. Interestingly, human CYP2C19 and marmoset CYP2C19 displayed enantiocomplementary selectivity in the hydroxylation of (*S*)‐ and (*R*)‐omeprazole, with the human enzyme hydroxylating the (*R*)‐enantiomer faster (Scheme 2, Tables S2–S5).

Similarly, CYP3A4, CYP2C19, CYP2D6, CYP2C8, CYP2C9, and their canine orthologues (e.g., canine CYP3A12 and canine CYP2D15) catalyzed the *N*‐dealkylation of (des‐*N*‐methyl) citalopram [67], sibutramine [60], fluoxetine [68], ketamine [69], lumefantrine [70], and verapamil [42] (Scheme 2). In addition, CYP2D6 catalyzes the O‐dealkylation of 3,4‐methylenedioxymethamphetamine (MDMA) [71, 72] and tramadol [73] with enantiopreference for the (*S*) or (*S, S*) enantiomers, respectively. Also, CYP3A4 catalyzes the *S‐*dealkylation of lesinurad [65], with preference for the (*S*)‐enantiomer. Other notable CYP‐catalyzed transformations include the dehydrogenation/aromatization of amlodipine [74] to the corresponding amlodipine pyridine derivative catalyzed by CYP3A4, CYP2C9, and CYP2C19, and the dehydration of doxazocin [59] by CYP2C8. The CYP enzymes were also associated with the racemization of rabeprazole and pantoprazole [75].

**SCHEME 3:**
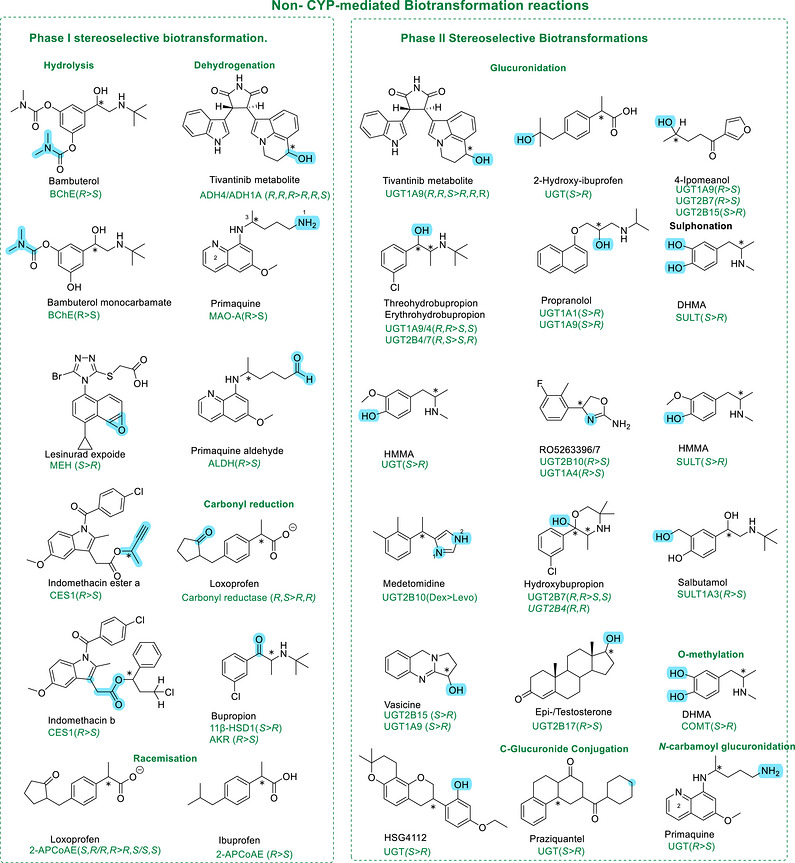
Non‐CYP‐mediated Phase I and II biotransformation reactions. Sites of reactions are indicated with color highlights. Stereopreference is indicated by the greater‐than symbol (>): for example, *S* > *R* means the (*S*)‐enantiomer is metabolized faster than the (*R*)‐enantiomer. 2‐APCoE, 2‐aryl propionyl‐coenzyme A epimerase; ADH, alcohol dehydrogenase; ALDH, aldehyde dehydrogenase; BChE, butyrylcholinesterase; CE, carboxylesterase; MDMA, 3,4‐methylenedioxymethamphetamine; MEH, microsomal epoxide hydrolases; SULT, sulfotransferase; UGT, UDP‐glucuronosyltransferase. See also Tables S2–S5.

Non‐CYP‐catalyzed reactions include carbamate hydrolysis of bambuterol and bambuterol monocarbamate catalyzed by butyrylcholinesterase (BChE) [76], epoxide hydrolysis of lesinurad epoxide catalyzed by microsomal epoxide hydrolase (MEH) [65], ester hydrolysis of indomethacin esters by carboxylesterase (CES1) [77], alcohol dehydrogenation of tivantinib metabolites catalyzed by human alcohol dehydrogenases, ADH4 and ADH1A [66], deamination of primaquine to the corresponding aldehyde by monoamine oxidase A (MAO‐A), and subsequent aldehyde oxidation by aldehyde dehydrogenase to the corresponding primaquine carboxylic acid metabolite [35], with carboxyprimaquine (CPQ) preferentially formed from the (−)‐PQ. Others include carbonyl reduction of bupropion [78] and exoprofen [79] catalyzed by aldo‐keto reductases (AKR)/11β‐HSD1 to the corresponding secondary alcohols (Scheme 3, Tables S2–S5).

The Phase I transformations generated alcohol, phenolic, free amino, thiol, and carboxylate groups, which can further undergo Phase II conjugation reactions. UGTs catalyzed the O‐glucuronidation of hydroxylated tivantinib [66], hydroxylated bupropion [80], 2‐hydroxy‐ibuprofen [64], vasicine [81], medetomidine [69], epi/testosterone [82], HSG4112 [83], 4‐ipomeanol [84], and HMMA [71, 72]. Similarly, SULT mediated the O‐sulfation to the catechol group of DHMA [71, 72], or the phenolic group of HMMA [71, 72], or benzyl alcohol of salbutamol [85], while O‐methylation of the catecholic group of DHMA is catalyzed by catechol‐O‐methyltransferase (COMT) [71, 72] to generate the methoxy derivatives, Scheme 3. Similarly, *N*‐conjugates, such as *N‐*glucuronidated R05263396/7, the *N*‐carbamoylglucuronide [86] of primaquine, were generated through UGT‐catalyzed *N*‐glucuronidation, and the C‐glucuronidation of praziquantel [87] was reported to be catalyzed by UGT (Scheme 3, Tables S2–S5).

The Phase II enzymes also displayed stereoselective metabolism. For example, a panel of UGTs screened against bupropion enantiomers and racemic bupropion revealed that UGT2B7 catalyzes the glucuronidation of (*S*,*S*)‐, (*S*,*R*)‐, and (*R*,*S*)‐hydrobupropion, whereas UGT1A9 catalyzes the glucuronidation of (*R*,*R*)‐hydrobupropion (Scheme 3). These findings were suggested to explain the variability in the therapeutic and toxicological effects of bupropion in humans [80]. Similarly, a major metabolite of primaquine (PQ) from the Phase I oxidative deamination pathway, carboxyprimaquine (CPQ), was formed preferentially from the (−)‐PQ, while the glycosylated conjugate of PQ on the terminal amine was preferentially generated with (+)‐PQ, and the *N*‐carbamoyl glucuronide of PQ was exclusively generated from (+)‐PQ [35].

In many cases, the differential metabolic rates of these enantiomers observed in vitro correlate with in vivo metabolite accumulation and clearance. For example, the butyrylcholinesterase (BChE)‐mediated hydrolysis of (*R*)‐bambuterol and (*R*)‐bambuterol monocarbamate was shown to be 4‐fold faster than that of the (*S*)‐enantiomer [76]. In a subsequent in vivo study, Tan and coworkers analyzed blood samples collected before administration and at multiple time intervals (0.25–96 h) after administration in 8 healthy volunteers who had been administered racemic bambuterol and its metabolites, including mono‐carbamate bambuterol and terbutaline. They observed that the *C*_max_ and AUC_0_–t of the (*S*)‐enantiomers were higher than those of the (*R)*‐enantiomers. This was attributed to the slower metabolic clearance rate of the (*S*)‐enantiomer compared to the (*R*)‐enantiomer of bambuterol [88]. The accumulation of (*S*)‐bambuterol may have toxicological implications, especially with racemic administration, as the (*S*)‐enantiomer (the less active isomer) has been associated with adverse cardiac toxic effects [89, 90]. Similar patterns of observations had been reported in a rat study; however, the differences in serum enantiomer concentrations were less pronounced than those observed in human studies, due to the poor stereoselectivity of rat BChE [91]. Likewise, extrapolating in vitro intrinsic clearance to in vivo human hepatic blood clearance, using the well‐stirred liver model, showed that the hepatic clearance rate of (+)‐PQ was only 45% that of (−)‐PQ. One of the major metabolic routes is the oxidative deamination of the terminal amine, leading to the formation of carboxyprimaquine aldehyde as the major metabolite, which was preferentially formed by the (−)‐PQ [35].

With growing recognition of the critical role of stereoselectivity in drug metabolism, there is an increasing need to map stereoselective properties across diverse drug libraries, and major DMEs, and subcellular fractions from different metabolic tissues. Additional data are also required to establish in vitro stereoselectivity patterns that reliably correlate with in vivo metabolite formation and clearance, thereby strengthening the physiological relevance of in vitro findings.

## 4 Differences in the Stereoselectivity of Commonly Used Subcellular Fractions

**SCHEME 4:**
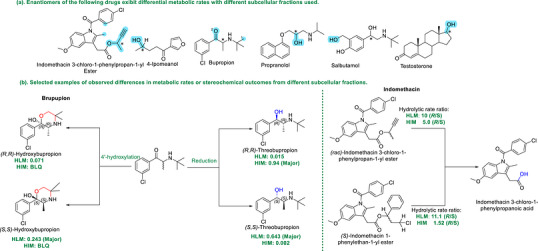
Differences in the stereoselectivity of subcellular fractions toward the biotransformation of selected drugs. BLQ, below the limit of quantification; HIM, human intestinal microsomes; HLM, human liver microsomes.

Our analysis showed distinct stereoselective properties of commonly employed subcellular fractions used in drug metabolism studies. Differences in enantiopreference or stereochemical outcomes were reported in the metabolism of 4‐ipomeanol, bupropion, indomethacin, propranolol, R0526336, salbutamol, and testosterone, depending on the type of subcellular fractions used (Table 1). For example, the ketone reduction of bupropion using HLM favored the formation of *(S,S)‐*threobupropion (THBUP), whereas *(R,R)‐*THBUP was preferentially formed with human intestinal microsomes (HIM) [69] (Scheme 4). Bamfo et al. suggested that 11β‐HSD was responsible for the metabolism of (*S*)‐bupropion in HLMs, whereas intestinal reduction of (*R*)‐bupropion was mediated by cytosolic carbonyl reductases and other enzymes [69]. In addition, enantiomers of 4‐hydroxybupropion (OHBUP) were detected only in liver subcellular fractions after in vitro incubation, with preferential formation of the (*S*,*S*)‐OHBUP enantiomer (Scheme 4). In contrast, negligible intestinal expression of CYP2B6 resulted in a lack of OHBUP detection [78].

**TABLE 1:** Differences in the stereoselectivity of subcellular fractions toward the biotransformation of selected drugs.

Entry	Chiral drug	Study design	Key findings	Ref.
1	Bupropion	In vitro Racemic and *(R)‐/(S)‐*forms incubated with pooled mixed‐sex HLM, HLS9, HLC, HIM, HIS9, and HIC.	4‐Hydroxybupropion (OHBUP) was only detected in HLM and HLS9. *(S,S)‐*OHBUP formation was greater than that of (*R*,*R)*‐OHBUP. HIM had the highest *(R,R)‐*THBUP formation of cellular subfractions. HLM showed the highest *(S,S)‐*THBUP formation of cellular subfractions.	[78]
2	4‐Ipomeanol	In vitro Racemic and *(R)‐/(S)‐*forms incubated with HLM and HKM.	*(R):(S)‐*ipomeanol glucuronide ratio was 57:43 (HLM) and 79:21 (HKM)	[84]
3	Indomethacin benzyl esters	In vitro *(R)‐/(S)‐*forms incubated with HLM and HIM.	Stereoselective differences in hydrolysis were observed only in HIM	[77]
4	Propranolol	In vitro *(R)‐/(S)‐*forms incubated in HLM, HIM, and HKM.	*(R)‐*propranolol was the preferential enantiomer in HLM and HKM, but not in HIM	[82]
5	R0526336/7	In vitro *(R)‐/(S)‐*forms incubated in HLM, HIM, and HKM.	*(R)‐*R0526337 was the preferential enantiomer in HLM but was not measurably detected in HIM or HKM.	[82]
6	Salbutamol	In vivo PO and inhaled racemic salbutamol and levosalbutamol administered (*n* = 1)	(*R*)‐Salbutamol was the preferential enantiomer, with a greater proportion of the dose metabolized after oral administration compared with inhalation.	[85]
7	Testosterone/epitestosterone	In vitro Individual epimers incubated in HLM and HKM	Testosterone: HIMs are 4‐fold more active in glucuronidation than HLMs. Epitestosterone: HLMs are 5‐fold more active in glucuronidation than in HIM.	[82]

Abbreviations: HIC, human intestinal cytosol; HIM, human intestinal microsomes; HIS9, human intestinal S9 fraction; HKM, human kidney microsomes; HLC, human liver cytosol; HLM, human liver microsomes; HLS9, human liver S9 fraction; OHBUP, 4‑hydroxybupropion; PO, per oral; THBUP, threohydrobupropion.

Similarly, kidney microsomes catalyzed the enantioselective glucuronidation of 4‐ipomeanol due to the expression of the *(R)‐*selective UGT1A9 in the kidney. In contrast, in the liver, poor stereoselectivity was observed due to the concurrent expression of two UGT isoforms, 2B7 and 2B15, which exhibit opposite stereoselectivities [84] (Table 1). Additionally, (*R*)‐propranolol was glucuronidated preferentially by UGT1A9 in the liver and kidney, compared to the intestine, as shown in Table 1 [82]. When drugs are administered via different routes, it is crucial to examine stereoselectivity at extrahepatic metabolic sites. For example, (*R*)‐salbutamol was more extensively sulfonated after oral administration than after inhalation, due to higher SULT1A3 expression in the jejunum than in the lung (Table 1) [85].

Carboxylesterases (CES) play crucial roles in activating ester‐type prodrugs [92]. CES1 and CES2 are expressed in the liver and intestine, respectively, and can both metabolize ester‐containing drugs. For example, after the incubation of indomethacin benzyl esters, stereoselective hydrolysis was only observed in the liver due to the expression of (*R*)‐selective CES1; in contrast, as suggested by Takahasi et al., the larger active site of CES2 was proposed to result in poor enantioselectivity for the indomethacin ester [77]. Differences in the stereoselectivity of subcellular fractions and the associated tissues can affect prodrug activation. There is currently considerable interest in the development of prodrugs that are activated by DMEs in vivo, especially in chemotherapy [93, 94, 95, 96]. Several enzyme‐mediated prodrug activation strategies have been employed, mainly using hydrolytic enzymes such as carboxylesterases [97, 98], proteases (e.g., caspase‐3 [99, 100] and cathepsin B [101]), β‐galactosidase [102, 103], and γ‐glutamyltranspeptidase. Investigating how these stereoselective enzymes influence chiral prodrug metabolism/activation in different tissues could further improve the effectiveness and safety of enzyme‐mediated prodrug activation strategies.

In vitro drug metabolism studies using subcellular fractions provide a framework for understanding the metabolic fates of drug candidates once administered. The distinct stereoselectivities associated with different tissues/cells highlight the importance of extensive multi‐organ or multi‐tissue in vitro metabolic studies. Enzyme expression patterns in different cells or tissues are the main contributors to the differential stereoselective properties of subcellular fractions. However, other factors, such as in vitro incubation conditions (e.g., pH, temperature, substrate and cofactor concentrations, and the percentage of organic cosolvents used); the source of subcellular fractions (including interindividual variability); the preparation of subcellular fractions; and the physiological state of cells, should be carefully considered when assessing and comparing stereochemical outcomes from in vitro biotransformation using different subcellular fractions. Jia and Liu have proposed a general framework to guide the design of in vitro drug metabolism studies [104, 105], and a harmonized framework for the conduct of in vitro metabolite identification has been proposed by Savaryn et al. [106]. If adopted, these frameworks could help reduce variability and improve the predictability and translational relevance of in vitro stereoselectivity.

## 5 Differences in the Stereoselective Properties of Orthologs of Drug Metabolizing Enzymes in Commonly Used Models

Drug metabolism studies in animal models are performed during preclinical drug development projects to assess both the metabolic stability and metabolic fate of a drug candidate, as well as to identify metabolites from in vivo biotransformations. Alongside toxicity studies in animals, drug metabolism studies in animal models ensure that the drug candidates being developed are safe for use in clinical trials. It is generally accepted that, with appropriate selection of animal models or orthologues of DMEs and well‑designed experimental approaches, metabolism data from animal studies for most drugs can be reasonably extrapolated to humans [107]. In extrapolating animal drug metabolism studies to humans, careful consideration is given to the possibility of interspecies differences in metabolite formation and metabolic pathways. However, these considerations are often not extended to potential differences in stereochemical outcomes or enantioselectivity across these species. This is a significant gap, especially since orthologs of DMEs may exhibit enantiocomplementary selectivity.

**SCHEME 5:**
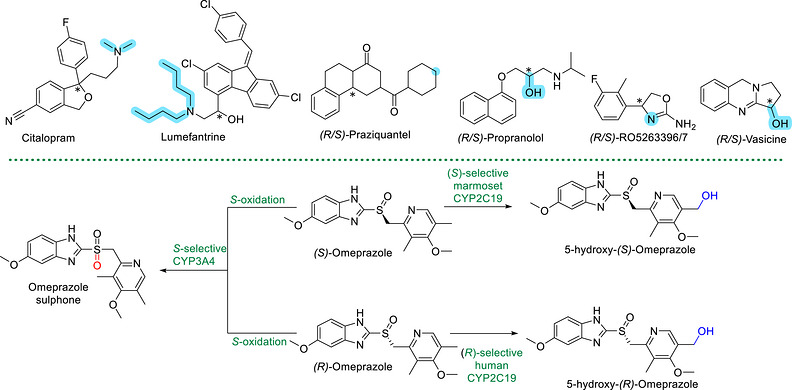
Species‑dependent stereoselective properties of orthologous drug‑metabolizing enzymes, exemplified by enantiocomplementary omeprazole 5‑hydroxylation.

Our analysis showed that 14% of the investigated chiral drugs showed species‐specific patterns in stereoselective metabolism as observed with citalopram, HSG4112, omeprazole, praziquantel, propranolol, RO5263396/7, and vasicine (Table 2). For example, humans, marmosets, and cynomolgus monkeys all express CYP2C19, a key enzyme that catalyzes the 5‐hydroxylation of omeprazole. While human and cynomolgus CYP2C19 were selective for *(R)*‐omeprazole 5‐hydroxylation, the marmoset CYP2C19 was selective for *(S)‐*omeprazole (Scheme 5). Uehara et al. noted that the omeprazole enantiomers docked similarly to CYP2C19 in humans and cynomolgus monkeys, whereas (*S*)‐omeprazole docked closer to the active site of the modeled marmoset CYP2C19, providing a molecular explanation for the observed species‑dependent stereoselectivity of omeprazole (Scheme 5) [108].

**TABLE 2:** Differences in the stereoselective properties of orthologs of drug metabolizing enzymes in commonly used models.

Entry	Chiral Drug	Study Design	Key Findings	Ref.
1	Omeprazole	In vitro Racemic and *(R)‐, (S)‐* enantiomers each incubated in liver microsomes from human, cynomolgus, and marmoset.	Marmoset CYP2C19 was selective for *(S)‐*omeprazole Human and cynomolgus CYP2C19 were selective for *(R)‐*omeprazole. Catalytic activity of recombinant CYP3A4 was conserved across non‐human primates and human.	[108]
2	HSG4112	In vivo and in vitro Oral and intravenous racemic form administered to rats and dogs. The *(R)‐* and *(S)‐*enantiomers were each incubated in liver microsomes from human, dog, and rat.	*(R)‐*HSG4112 was preferentially metabolized in rats, whereas *(S)‐*HSG4112 was preferentially metabolized in dogs. HSG4112 metabolism was CYP‐mediated in rats and humans but UGT‐mediated in dogs.	[83]
3	Praziquantel	In vitro *(R)‐/(S)‐* forms incubated in liver and intestinal microsomes from humans and mice.	HLM showed reduced production of mono‐oxidized praziquantel metabolites compared with MLM. HIM displayed higher catalytic activity toward praziquantel compared with MIM.	[87]
4	Propranolol	In vitro Racemic, *(R)‐*, and *(S)‐*enantiomers were each incubated in liver microsomes from humans, mice, rats, dogs, minipigs, and monkeys.	Only monkeys and mice exhibited selectivity for (*R*)‐propranolol similar to humans. Rats and minipigs displayed *(S)‐*propranolol selectivity.	[82]
5	R0526336/7	In vitro Racemic, *(R)‐, (S)‐*enantiomers incubated in liver microsomes of human, mouse, rat, dog, minipig and monkey.	Mouse, rat, dog, minipig, and cynomolgus monkey microsomes exhibited selectivity for (*R*)‐R0526337 glucuronidation	[82]
6	Vasicine	In vitro Racemic, *(R)‐, (S)‐*enantiomers each incubated in liver microsomes from humans, rats, mice, pigs, guinea pigs, dogs, monkeys, rabbits, bulls, sheep, and camels.	Preferential *(S)‐*vasicine glucuronidation was significantly greater in guinea pig microsomes compared to HLM. Rat, mouse, pig, dog, monkey, rabbit, bull, sheep, and camel microsomes showed only slightly faster preferential *(S)‐*vasicine glucuronidation compared with HLM.	[81]
7	Citalopram	In vitro Racemic and *(R)‐, (S)‐*enantiomers are incubated in HLM and DLM.	Selective for *(S)‐*citalopram. Canine CYP2D15 showed 10‐fold higher affinity for citalopram and 100‐fold higher affinity for DCIT compared with human CYP2D6 despite sharing significant homology. DDCIT formation was significantly higher in dogs than in humans.	[67]

Abbreviations: CLM, cynomolgus monkey liver microsomes; CYP, cytochrome P450; DCIT, N‑desmethylcitalopram; DLM, dog liver microsomes; HLM, human liver microsomes; MLM, mouse liver microsomes; MpLM, minipig liver microsomes; RLM, rat liver microsomes; UGT, UDPglucuronosyltransferase.

Bae et al. administered racemic HSG4112 orally and intravenously to both rat and dog models [83]. In the rat model, *(R)‐*HSG4112 was more extensively metabolized, whereas the opposite stereoselectivity was reported in the dog model [83]. The authors suggested that HSG4112 metabolism was primarily CYP‐mediated in rats, whereas in dogs high glucuronidation activity was observed, with a preference for *(S)‐*HSG4112 [83] (Table 2). The rat model was therefore more accurate in predicting the stereoselective metabolism of HSG4112 in humans. Species‐dependent stereoselectivity is not limited to Phase I metabolism, as, following incubation with liver microsomes of various species, *(S)‐*RO5263397 had greater UGT2B10‐ and UGT1A4‐mediated glucuronidation than its enantiomer, *(R)‐*RO5263396 [82]. Additionally, after incubating propranolol with liver microsomes of multiple species, only cynomolgus monkeys and mice showed selectivity similar to humans for (*R*)‐propranolol UGT1A9‐catalyzed glucuronidation, whereas rats and minipigs exhibited selectivity for *(S)‐*propranolol [82] (Table 2).

Species‐specific differences in stereoselectivity underscore the need to carefully select appropriate preclinical animal models for drug metabolism studies to avoid misleading extrapolations of stereoselective metabolism pathways in humans. The study conducted by Milani et al. thoroughly investigated stereoselective metabolic profiles of 4 chiral drugs in 5 different animal species to human‐derived in vitro systems [82]. Rigorous drug metabolism studies such as this would help bridge this translational gap to ensure more reliable preclinical assessments.

Early in vivo ADME studies are crucial in reducing the attrition rate of newly developed drugs. Interspecies variation can lead to differences in the toxicokinetic profiles of chiral drugs, as different enantiomers may be preferentially metabolized in different species, complicating the results of preclinical toxicity assessments. For example, citalopram was incubated in HLM and beagle dog liver microsomes (Table 2). Canine CYP2D15 is an ortholog of human CYP2D6, and both catalyze (*S*)‐selective citalopram *N*‐demethylation [67]. The isozyme affinity for citalopram was 10‐fold higher and at least 100‐fold higher for *N*‐desmethyl‐citalopram (DCIT) in CYP2D15 compared to CYP2D6 [67]. *N*‐didesmethyl‐citalopram (DDCIT) formation was higher in dogs [67]. Significantly elevated DDCIT concentrations can cause QTc prolongation and an increased risk of arrhythmia. Hypothetically, based on the in vivo metabolic data from beagle dogs, the development of citalopram could have been prematurely halted before human clinical studies, which revealed that DDCIT production was a minor pathway in humans.

Significant challenges persist in predicting human stereoselective metabolism data from animal models. Although orthologs of specific CYP isoforms have been characterized, further research is needed to establish equivalence among the orthologs of other major drug‐metabolizing enzymes (e.g., UGT) [57]. One strategy to mitigate interspecies variability is the use of humanized animal models, which can provide more human‐relevant metabolic profiles. In applying these humanized models, residual murine enzymatic activities can compromise correlation with human in vivo data. To address this limitation, multiple groups have engineered refined models with selected murine genes removed. For example, Wolf et al. developed a humanized mouse, 8HUM, by replacing 33 murine CYPs with human CYP1A1, CYP1A2, CYP2C9, CYP2D6, CYP3A4, and CYP3A7 isoforms, and by substituting murine constitutive androstane receptor (CAR) and pregnane X receptor (PXR) with their human counterparts [52]. Using the 8HUM model, they investigated drug metabolite profiles of 14 drugs and compared biotransformation results with human liver microsomes, demonstrating that 8HUM exhibits a humanized drug metabolism profile [52]. The 8HUM model has also been used to investigate drug–drug interactions involving the peptide NN1177 and small‐molecule drugs [53].

Similarly, Uehara et al. have developed multiple chimeric versions of humanized‐liver TK‐NOG mice [54, 109, 110], including a model with a near‐complete knockout of liver‐specific cytochrome P450 oxidoreductase (POR cKO Huliver) to minimize background oxidation from murine hepatic CYPs [54] and another model in which the murine carboxylesterase 1c gene (*Ces1c*) was deleted. The *Ces1c* knockout humanized‐liver mouse was used to compare hydrolytic products and CYP3A‐mediated oxidation of irinotecan, oseltamivir, and temocapril, revealing good correlation with human data [109]. This *Ces1c* knockout model was proposed as a platform for studying human CES1‐mediated metabolism, as it retains human‐like plasma esterase and hepatic drug metabolizing activities.

Yokoi et al. demonstrated that chimeric mice transplanted with human hepatocytes (uPA^+/+^/SCID mice) displayed metabolic profiles comparable to humans. This model expressed sufficient levels of human CYP2D6 and showed a humanized metabolic profile toward debrisoquin, a model CYP2D6 probe [55]. Karlsson et al. examined drug metabolite profiles resulting from the biotransformation of 12 drugs in chimeric PXB‐mice, whose livers are engrafted with up to 95% human hepatocytes. They reported that Phase I reactions, including hydroxylations and O‐ and *N‐*dealkylations, as well as a Phase II glucuronidation reaction, produced metabolic profiles comparable to those observed in human in vivo studies [111].

As humanized models become increasingly important for in vivo drug metabolism studies, their utility should be extended to investigations of the stereoselectivity of enzymes expressed in these systems and the stereochemical outcomes of chiral metabolites generated within them.

## 6 Effects of Genetic Polymorphism and Chiral Inversion on Stereoselective Drug Metabolism

Polymorphisms in genes encoding some DMEs are a key source of interindividual and interethnic variability in biotransformation efficiencies [112, 113]. For a given gene or biotransformation pathway, individuals or populations may be categorized as poor, intermediate (or normal), or extensive/ultra‐rapid metabolizers. These metabolic phenotypes are often linked to ethnic background [114]. Understanding these polymorphisms is particularly important in personalized medicine, as it can assist in selecting appropriate treatment regimens to improve therapeutic outcomes or avoid adverse reactions. The metabolism of several chiral drugs, including efavirenz, fluoxetine, omeprazole, tivantinib, and warfarin, is affected by polymorphisms in cytochrome P450 (CYP) enzymes. While interindividual and interethnic variations in the metabolic profiles of drugs affected by CYP genetic polymorphisms have been well studied, the stereochemical aspects of these variations remain comparatively underexplored.

**SCHEME 6:**
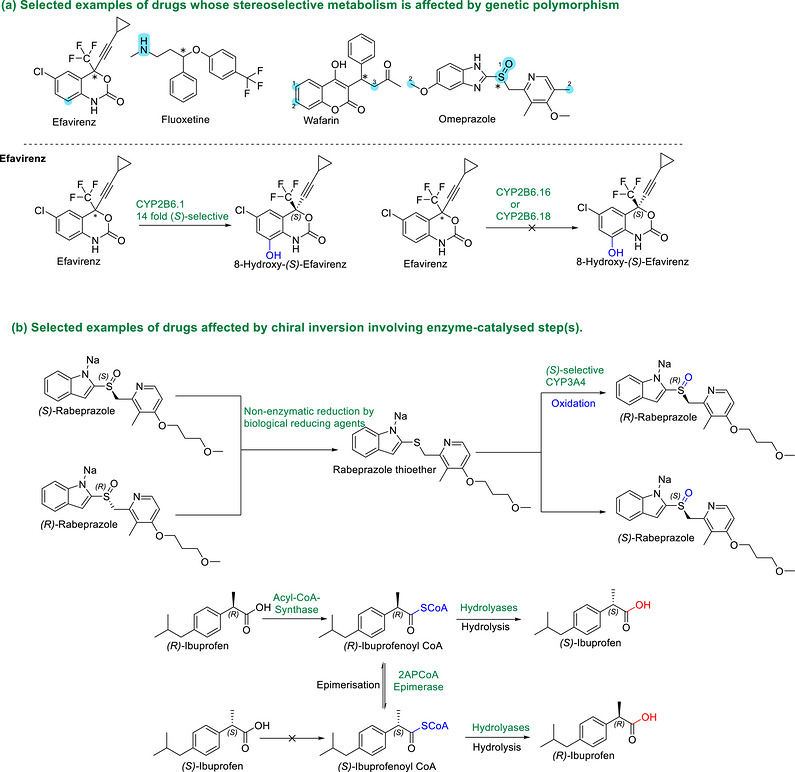
Effects of genetic polymorphism and chiral inversion on stereoselective drug metabolism with examples of drugs affected. 2APCoA, 2‐arylpropionyl‐coenzyme A.

The primary metabolic route of the antiretroviral agent efavirenz is CYP2B6‐catalyzed hydroxylation to 8‐hydroxyefavirenz. Wang et al. expressed human recombinant wild type CYP2B6 and 10 variants in insect cells and incubated each variant with individual efavirenz enantiomers, revealing a clear preference for the metabolism of (*S*)‐efavirenz over the (*R*)‐enantiomer (Table 3, Scheme 6). However, the relative catalytic activity varied substantially among the variants. While CYP2B6.4 and the wild type exhibited (*S*)‐selective 8‐hydroxylation, the CYP2B6.16 and CYP2B6.18 variants showed only weak activity [115]. Wang et al. further showed that alleles *6, *9, *16, and *18 cause loss‐of‐function for (*S*)‐efavirenz 8‐hydroxylation, resulting in a poor metabolizer phenotype (Table 3). Notably, the allelic patterns (CYP2B6 *6/*6 and CYP2B6 *6/*18) are prevalent among Africans or African Americans [115]. These differences in stereoselective clearance and metabolism may therefore lead to substantial variability in therapeutic efficacy and safety profiles across ethnic groups.

**TABLE 3:** Influence of genetic polymorphisms and chiral inversion on stereoselective drug metabolism.

Entry	Chiral drug	Study design	Key findings	Ref.
**Genetic polymorphism**				
1	Efavirenz	In vitro Racemic, *(R)‐, (S)‐*enantiomers each incubated with human CYP2B6 recombinant CYP2B6 variants expressed in *Spodoptera frugiperda* (Sf9) and *Trichoplusia ni* cells CYP2B6 variants investigated: wild type 2B6.1, 2B6.4, 2B6.5, 2B6.6, 2B6.7, 2B6.9, 2B6.17, 2B6.19, 2B6.26.	Efavirenz metabolism was highly selective for (S)efavirenz, exhibiting a 14‐fold preference over (R)efavirenz. CYP2B6.1 exhibited the highest (S)efavirenz 8‐hydroxylation, whereas CYP2B6.16 and CYP2B6.18 were inactive. CYP2B6.4 displayed the highest (R)efavirenz hydroxylation activity, followed by CYP2B6.17, CYP2B6.5, and CYP2B6.1.	[115]
2	Fluoxetine	In vitro Racemic, *(R)‐, (S)‐*enantiomers each incubated with human recombinant CYP2C9 variants expressed in Sf9 cells CYP2C9 variants investigated: wild type 2C9.1, 2C9.3, 2C9.13, and 2C9.16.	At concentrations > 100 µM, a stereoselective self‐inhibitory effect was observed in CYP2C9.1‐mediated *N*‐demethylation. No stereoselective inhibitory effect was observed for CYP2C9.3, CYP2C9.13, or CYP2C9.16.	[68]
3	Omeprazole	In vitro Racemic, *(R)‐, (S)‐*enantiomers each incubated with cynomolgus liver microsomes. CYP2C19 allelic patterns investigated: wild type, heterozygous, and homozygous.	*(R)‐*omeprazole 5‐hydroxylation was significantly affected by allelic patterns, but *(S)‐*omeprazole 5‐hydroxylation was not. *(R)‐*omeprazole 5‐hydroxylation was lowest in the homozygous group compared with the wild type.	[108]
4	Warfarin	In vivo 10 mg oral warfarin administered to genotyped volunteers. CYP2C9 allelic patterns investigated: 2C9*1/*1 (*n* = 8), 2C9*1/*3 (*n* = 9), 2C9*2/*3 (*n* = 3), 2C9*3/*3 (*n* = 4).	Metabolism was selective for *(S)‐*warfarin *(S)‐*warfarin hydroxylation was impaired with the increasing number of defective *2 and *3 CYP2C9 variant allele. *(R)‐*warfarin hydroxylation was unaffected by CYP2C9 genotype.	[62]
**Chiral inversion**				
5	Rabeprazole	In vivo and In vitro *(R)‐, (S)‐*Rabeprazole thioether intravenously administered to rats *(R)‐, (S)‐*rabeprazole thioethers incubated with HLM.	Higher plasma concentrations of rabeprazole thioether in the portal vein compared with systemic circulation. Rabeprazole thioether was oxidized to form the sulfoxide group of the parent drug. Plasma concentrations of *(R)‐*rabeprazole were significantly higher than those of *(S)‐*rabeprazole. In HLM, the thioether was preferentially metabolized to *(R)‐*rabeprazole.	[75]
6	Ibuprofen	In vivo Racemic, *(R)‐, (S)‐*enantiomers each intravenously administered to rats.	*(R)‐*ibuprofen was not detected in plasma after *(S)‐*ibuprofen administration. *(S)‐*Ibuprofen was detected in plasma after *(R)‐*ibuprofen administration, demonstrating in vivo chiral inversion of *(R)‐* to the *(S)‐*enantiomer.	[64]

Abbreviations: CYP, cytochrome P450; DCIT, N‐desmethylcitalopram (citalopram metabolite); DDCIT, N‐didesmethylcitalopram (double N‐demethylated citalopram metabolite); IV, intravenous; PM, poor metabolizer phenotype; Sf9 cells, *Spodoptera frugiperda* insect cells; T. ni cells, *Trichoplusia ni* insect cells; WT, wild type.

The metabolism of *(S)‐*warfarin to its 6‐hydroxymetabolite is catalyzed by CYP2C9. Flora et al. administered 10 mg of warfarin orally to healthy human volunteers and showed that its metabolism was stereoselective for *(S)‐*warfarin. The difference in plasma concentration ratios for the enantiomers *(S)‐*warfarin*:(R)‐*warfarin between CYP2C9 *3/*3 and wild type *1/*1 was 1.09 and 2.81 at 2 and 48 h after administration, respectively (Table 3) [62]. While metabolism was stereoselective for *(S)‐*warfarin, and CYP2C9‐mediated metabolism was impaired with an increasing number of defective CYP2C9 variant alleles, *2 and *3, whereas *(R)‐*warfarin metabolism remained unaffected by the CYP2C9 allelic pattern [62] (Table 3).

The in vivo genetic studies reviewed were ethnically skewed, which limits the generalizability of their findings across diverse populations. Moreover, genotype alone may not fully account for observed stereoselectivity; the contributions of epigenetic regulation, disease state, and drug–drug interactions (DDIs) to stereodivergent metabolism should also be considered. Incorporating complementary in vitro approaches, such as engineered recombinant enzyme systems and additional site‑directed mutagenesis studies, could help validate in vivo findings and provide mechanistic insight. Although CYP variants are reasonably well characterized with respect to their influence on stereoselective drug metabolism (SDM), other enzymes such as UGTs and SULTs remain comparatively under‑investigated.

*Enzyme‐Mediated Chiral Inversion*. The enantiomer of a chiral drug may be converted to the opposite enantiomer in vivo via chiral inversion, potentially affecting the drug's therapeutic profile and toxicity. Non‑enzymatic processes can drive chiral inversion as a drug moves between biological compartments due to changes in physicochemical conditions or the presence of endogenous reducing agents. However, a substantial proportion of these processes involves a combination of non‑enzymatic and enzyme‑catalyzed transformations. In some cases, chiral inversion occurs through a multi‑step enzyme‑catalyzed pathway (Scheme 6).

For example, the mechanism for the chiral inversion of proton pump inhibitors (e.g., rabeprazole, omeprazole, lansoprazole, pantoprazole) was elucidated by Tang et al. to involve initial non‐enzymatic reduction to thioether intermediates by biological reducing agents in the liver, such as reduced glutathione and cysteine. The thioether metabolites are then enzymatically oxidized in a stereoselective fashion [65] (Table 3, Scheme 6). Ikuta et al. also proposed that the inactive (*R*)‐enantiomer of ibuprofen is converted to (*R)‐*ibuprofenyl‐adenylate, which is subsequently activated by acyl‐coenzyme A synthase to form (*R*)‐ibuprofen coenzyme A thioester. This thioester is then racemized by 2‐aryl propionyl‐coenzyme A epimerase and released as *(S)‐*ibuprofen by a hydrolytic enzyme [64] (Scheme 6, Table 3). Comprehensive mapping of the substrate specificities and enantioselectivities of enzyme systems frequently involved in chiral inversion can therefore guide the design of drug candidates that are less susceptible to chiral inversion.

## 7 Stereoselective Inhibition and Effect of Co‐Administered Drugs on Stereoselectivity

**SCHEME 7:**
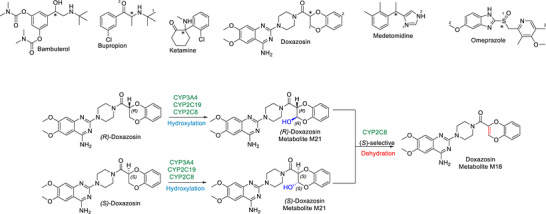
Examples of drugs whose metabolism is affected by stereoselective inhibition and effect of drug–drug interaction‐mediated effects on stereoselective metabolism.

Several drugs inhibit the metabolism of other drugs, and their concomitant administration can lead to clinically important drug‐drug interactions (DDIs). For example, among the chiral drugs reviewed, amlodipine, bambuterol, bupropion, doxazosin, ketamine, medetomidine, and omeprazole were identified as inhibitors or inducers of DMEs (Table 4, Scheme 7).

**TABLE 4:** Effects of drug–drug interactions on stereoselective drug metabolism.

	Chiral drug	Study design	Key findings	Ref.
1	Doxazosin	In vitro (*R*)‐, (*S*)‐enantiomers each incubated in rat liver microsomes In vivo Racemic form administered intravenously to Sprague–Dawley rats	After IV administration, (S)‐doxazosin was metabolized to a greater extent than (R)‐doxazosin. In separate microsomal incubations, (S)‐doxazosin did not deplete faster than (R)‐doxazosin.	[116]
2	Amlodipine	In vitro Racemic, *(R)‐, (S)‐*enantiomers each incubated in HLM with midazolam (CYP3A substrate), diclofenac (CYP2C19 substrate) and (*S*)‐mephenytoin (CYP2C19 substrate)	After 3min preincubation, (S)‐amlodipine inhibited midazolam hydroxylation more strongly than (R)‐amlodipine via a competitive mechanism. After 30 min preincubation, stereoselective midazolam inhibition was abolished. (R)‐amlodipine inhibited diclofenac 4′hydroxylation twofold relative to (S)‐amlodipine via a mixed inhibition model. (R)‐amlodipine inhibited (S)‐mephenytoin 4′hydroxylation 12‐fold compared with (S)‐amlodipine via a noncompetitive mechanism.	[74]
3	Bambuterol	In vitro Racemic, *(R)‐, (S)‐*enantiomers each incubated in physiological concentrations of butyrylcholinesterase (BChE).	Bambuterol and its monocarbamate transiently inhibit BChE before hydrolytic terbutaline formation. Selective for *(R)‐*bambuterol and *(R)‐*monocarbamate.	[76]
4	Bupropion	In vivo Single 100 mg dose of bupropion was administered to volunteers (*n* = 53), either alone or with a single 600 mg efavirenz dose, or following 17 days efavirenz pretreatment.	Acute efavirenz dosing inhibited CYP2B6‐mediated *(R)‐/(S)‐*bupropion hydroxylation, whereas chronic dosing induces it. Acute efavirenz dosing inhibited preferentially *(S, S)‐*threohydrobupropion formation.	[118]
5	Ketamine	In vitro Racemic ketamine incubated in dog and human liver microsomes.	*(S)‐*Ketamine inhibited the CYP3A12‐mediated *N*‐demethylation of *(R)‐*ketamine.	[69]
6		In vitro Racemic, (R)‐, (S)‐ enantiomers each incubated with recombinant human CYP2B6.	Formation of (*R*)/(*S*)‑norketamine was lower from the racemate than from the individual enantiomers.	[117]
7	Medetomidine	In vitro Racemic and dexmedetomidine incubated in HLM with ketamine	Levomedetomidine was the more potent enantiomer at low ketamine concentrations, resulting in stronger inhibition of (*R*)‐ketamine CYP3A12‐mediated *N*‐demethylation for racemic medetomidine than dexmedetomidine alone No stereoselective inhibition was observed for human CYP3A4, although induction of ketamine CYP3A4‐mediated *N*‐demethylation occurred at low dexmedetomidine concentrations.	[69]
8	Omeprazole	In vitro Racemic, *(R)‐, (S)‐*enantiomers each incubated with recombinant human, cynomolgus, and marmoset CYP2C19 with ticlopidine (CYP2C19 substrate)	Ticlopidine preferentially inhibited (R)‐omeprazole 5‐hydroxylation for human and cynomolgus CYP2C19, whereas (S)‐omeprazole 5‐hydroxylation was preferentially inhibited for marmoset CYP2C19.	

Abbreviations: BChE, butyrylcholinesterase; HLM, human liver microsomes; IV, intravenous.

Enantiomer‐enantiomer interactions can also occur when one enantiomer inhibits the metabolism of the other. Kong et al. reported that the plasma levels of *(S)‐*doxazosin were significantly lower than those of *(R)‐*doxazosin after racemic doxazosin was intravenously administered to Sprague‐Dawley rats [116]. However, in separate rat liver microsome incubations*, (S)‐*doxazosin did not deplete more rapidly than *(R)‐*doxazosin (Table 4) [116]. Kong et al. speculated that this discrepancy arose from stereoselective competitive inhibition of CYP3A4‐mediated (*S*)‐doxazosin metabolism, as (*R*)‐doxazosin binds more strongly to the active site of CYP3A4, thereby preventing *(S)‐*doxazosin from binding (Scheme 7).

Ketamine was identified as a chiral drug with numerous reported drug–drug interactions that altered either its own SDM or that of a co‐administered drug. Sanbaumhuter et al. showed that canine CYP3A12 catalyzed the *N*‐demethylation of (*S*)‐ketamine faster than that of (*R*)‐ketamine; however, racemic ketamine displayed substrate inhibition of CYP3A12‐mediated *N*‐demethylation [69]. This enantiomeric self‐inhibition was also reported by Wang et al. for ketamine incubations with recombinant human CYP2B6; they found that the rate of formation of (*R*)‐/(*S*)‐norketamine was lower from the racemate than for the individual enantiomers [117].

Stereoselective metabolism of ketamine was also affected by co‐administered drugs. Sanbaumhuter et al. reported that medetomidine binds to the CYP heme iron and acts as a competitive inhibitor, thereby inhibiting the rate of ketamine *N*‐demethylation. More potent inhibition was observed for *N*‐demethylation of *(R)‐*ketamine by canine liver microsomes and canine recombinant CYP3A12, whereas no stereoselectivity was observed with human CYP3A4 [69]. Similarly, the (*S*)‐enantiomer of S002‐333 ([2‐(4‐methoxy‐benzene sulfonyl)‐2,3,4,9‐tetrahydro‐1H‐b‐carboxylic acid amide) was found to be a potent inhibitor of CYP2B6‐catalyzed 6‐hydroxylation of bupropion, while the (*R*)‐enantiomer only showed weak inhibitory activity on CYP2B6 [119].

Amlodipine can act as a stereoselective competitive, non‐competitive, and mixed inhibitor. For example, after a 3‐min pre‐incubation period in HLMs, *(S)‐*amlodipine inhibited midazolam hydroxylation to a greater extent than (*R*)‐amlodipine via a competitive mechanism. However, after 30 min, stereoselectivity in midazolam inhibition was abolished, indicating that the inhibition was competitive, reversible, and time‐dependent [74]. In contrast, *(R)‐*amlodipine inhibited diclofenac 4′‐hydroxylation twice the level of inhibition observed for the (*S*)‐amlodipine, via a mixed inhibition mechanism, while *(R)‐*amlodipine inhibited CYP2C19‐mediated *(S)‐*mephenytoin 4′‐hydroxylation 12‐fold relative to *(S)‐*amlodipine via non‐competitive inhibition [74].

Genetic polymorphisms also influenced the DDIs observed. In one study, racemic fluoxetine and its *(R)‐* and *(S)‐*enantiomers were each incubated with human CYP2C8 and CYP2C9 recombinant proteins expressed in insect cells (Table 4) [68]. CYP2C8 showed significant selectivity toward *N*‐demethylation of *(R)‐*fluoxetine, with the variant CYP2C8.3 exhibiting the highest activity, followed by wild type CYP2C8.1, CYP2C8.2, and CYP2C8.4 [68]. Above 100 µM, a stereoselective self‐inhibitory effect was observed in CYP2C9‐mediated *N*‐demethylation, with the inhibition effect of (*R*)‐ to (*S*)‐fluoxetine being more potent than that of *(S)‐* to *(R)‐*fluoxetine in CYP2C9.1 [68]. Wang et al. reported that this stereoselective inhibition effect was not observed for CYP2C9.3, CYP2C9.13, and CYP2C9.16 genotypes (Table 4) [68].

In another study, 53 healthy volunteers received a single 100 mg dose of bupropion alone, a single 600 mg dose of efavirenz, or bupropion following a 17‑day pretreatment with efavirenz (600 mg day^−^^1^) [118]. Gufford et al. determined that chronic administration of efavirenz resulted in substantially higher metabolic ratios of *(S,S)‐*OHBUP/‐buproprion and *(R,R)‐*OHBUP/‐buproprion in normal metabolizers (CYP2B6*1/*1) and intermediate metabolizers (CYP2B6 *1/*6), whereas CYP2B6 activity was not induced in poor metabolizers [80]. Acute administration of efavirenz increased exposure of (*S,S*)‐THBUP but not its opposite enantiomer, (*R,R*)‐THBUP, with a greater effect observed in intermediate and poor metabolizers, as efavirenz inhibited (*S,S*)‐THBUP elimination in a CYP2B6 genotype‐ and concentration‐dependent manner [118] (Table 4).

DDIs can alter the stereoselective metabolic profile of chiral drugs, leading to slower metabolism and clearance of individual enantiomers, with potential clinical consequences for patients. More in‐depth pharmacokinetic studies are therefore required to characterize how DDIs influence stereoselective metabolism and to assess the clinical importance of such effects. These interactions complicate the design and interpretation of conventional DDI studies. Regulatory guidance may need to explicitly address the potential for enantioselective DDIs in preclinical and clinical R&D to ensure that interaction risks for chiral drugs are not underestimated.

## 8 General Discussion

### 8.1 Biocatalysis to Drive Sustainable Stereoselective Metabolite Synthesis

One of the major challenges in drug development is obtaining drug metabolites in quantities sufficient for comprehensive testing, characterization, and biological evaluation. Sustainable (bio)synthetic approaches are needed to access individual metabolite enantiomers to enable the assessment of their therapeutic and toxicological properties. However, the structural complexity of many drug candidates makes regio and stereoselective modification using conventional chemo(catalytic) methods particularly difficult. Subcellular fractions and recombinant enzymes offer effective strategies to overcome these limitations. Recombinant enzymes provide a selective and sustainable platform for metabolite synthesis. Recent advances have enabled the identification and engineering of ancestral variants of human DMEs, yielding more stable systems for metabolite production. When expressed in bacterial hosts, membrane‐bound preparations containing cytochrome P450 enzymes have proven to be robust and stable alternatives to human liver microsomes and recombinant human enzymes for both metabolite synthesis and metabolic pathway elucidation [120].

Gillam and collaborators at AstraZeneca identified thermostable ancestral CYPs and recombinantly expressed these enzymes in bacterial host cells. The membrane‐bound preparations, analogous to liver microsomes, were comparatively profiled for the metabolism of several drugs [120]. These ancestral CYPs showed similar profiles, and in some cases, metabolites not previously observed in humans or animal models could be synthesized using these platforms. A similar concept was applied to generate CYP3A4 ancestral proteins with enhanced thermostability and solvent tolerance [121]. Gillam and colleagues have also developed an enzyme library construction method based on ancestral sequence reconstruction, termed CLADE (combinatorial libraries of ancestors for directed evolution), and have deployed it to improve the thermostability of several enzymes, including CYP2D [121, 122].

**SCHEME 8:**
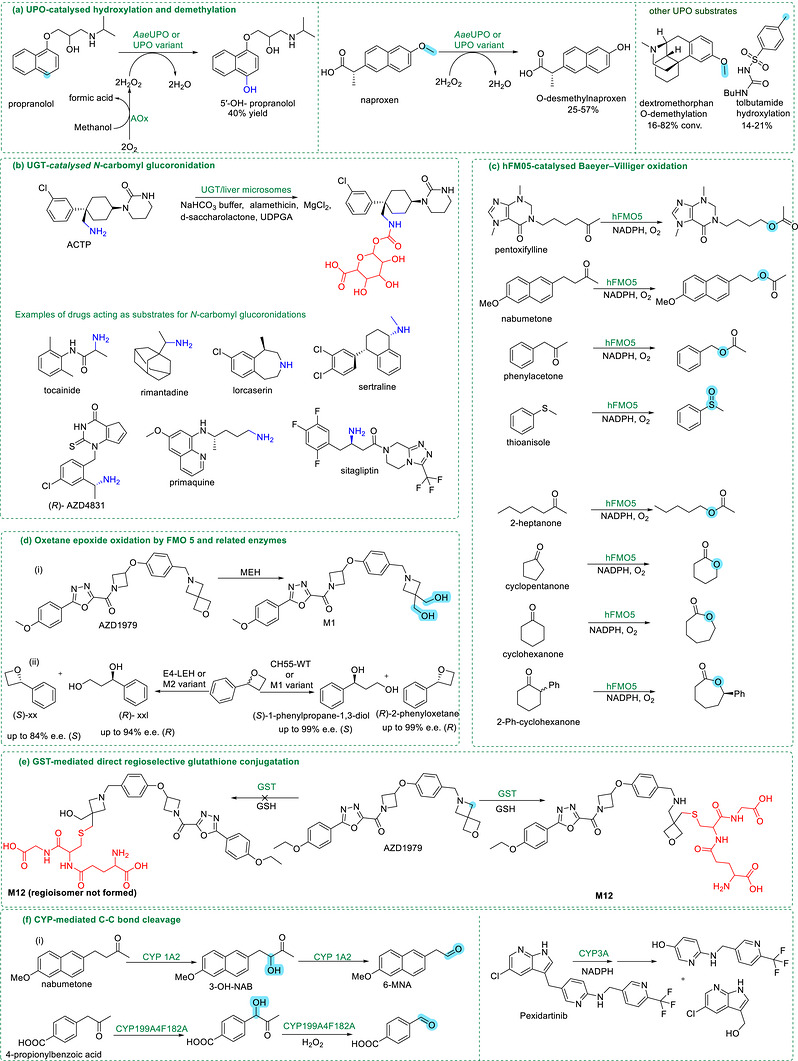
Selected examples of rare biotransformation reactions and the enzymes that catalyze them. CYP, cytochrome P450; GST, glutathione S‐transferase; hFMO, human flavin‐containing monooxygenase; LEH, limonene epoxide hydrolase; MEH, microsomal epoxide hydrolase; UGT, UDP‐glucuronosyltransferase; UPO, unspecific peroxygenase.

Similarly, Alcalde and coworkers investigated whether recombinant fungal unspecific peroxygenases (UPOs) could catalyze highly regioselective hydroxylation and O‐methylation to produce human CYP‐like metabolites. They screened a fungal UPO from *Agrocybe aegerita* (AaeUPO) and its evolved variants, expressed in *Saccharomyces cerevisiae* or *Pichia pastoris* expression systems, and screened against four model drugs: propranolol, dextromethorphan, naproxen, and tolbutamide (Scheme 8a), with an in situ H_2_O_2_  regeneration system [123, 124]. These enzymes generated the authentic human CYP metabolites with up to 82% conversion (Scheme 8a). UPOs are attractive biocatalysts for metabolite synthesis and in biocatalysis, as they do not require expensive nicotinamide cofactors and utilize H_2_O_2_ for their oxyfunctionalization [123, 124]. Grogan and Unsworth have further employed UPOs (artUPO and *Aae*UPO variants) for enantioselective oxygenation of sulfilimines and sulfenimines, enabling kinetic resolution to generate enriched sulfoximines and sulfinimines with up to 98% ee [125].

In some cases, bacterial homologues with often soluble expression and enhanced stability are usually employed in metabolite synthesis, such as soluble bacterial P450‐BM3 [126, 127, 128]. Recent examples of drug metabolites synthesized enzymatically are highlighted by Shanu‐Wilson et al. [129]. Several other non‐homologous isofunctional enzymes, not necessarily involved in human drug metabolism, have been identified and often engineered to devise biocatalytic routes to key metabolites [123, 129, 130]. In some cases, recombinant bacterial whole cells or bacterial cultures are employed as biocatalysts for scalable metabolite synthesis [130, 131, 132].

### 8.2 Drug Metabolism as a Source of Novel Biocatalysts and Inspiration for Novel Biomimetic Synthesis

Decades of drug metabolism studies have uncovered a broad spectrum of biotransformation reactions and a wide range of drug metabolizing enzymes (DMEs). Nonetheless, novel metabolites and metabolic pathways continue to be discovered, and some of these transformations are considered atypical. In a series of Reviews, Isin and Guengerich have documented several examples of such atypical biotransformation reactions [133, 134, 135]. These rare transformations can inspire the development of novel biocatalytic and biomimetic synthesis methods. DMEs mediating such reactions can therefore be exploited and further developed as novel biocatalysts for API and metabolite synthesis. Here, we highlight selected examples that have inspired synthetic applications.

UDP‐glycosyltransferases (UGTs) have been shown to mediate *N‐*carbamoyl glucuronidation of amines and amino acids. This transformation involves the reaction of primary or secondary amines with carbon dioxide to generate the corresponding carbamates [136], which are subsequently glucuronidated, yielding *N*‐carbamoyl glucuronides. Several in vitro studies have demonstrated that liver microsomes from various species, including rats, hamsters, dogs, monkeys, mice, and humans, catalyze this reaction. Gunduz et al. performed biotransformation reactions using microsomes or UGT supersomes incubated with NaHCO_3_, an amine‐containing substrate (1‐[4‐aminomethyl‐4‐(3‐chlorophenyl)‐cyclohexyl]‐tetrahydro‐pyrimidin‐ 2‐one, ACTP), alamethicin (to remove latency in UGT activity and increase substrate permeability), MgCl_2_, D‐saccharolactone, and UDPGA. UGT1A3, UGT1A1, and UGT2B7 supersomes were shown to catalyze *N*‐carbamoyl glucuronidation [137]. Similarly, Sadeque et al. found recombinant UGT2Bs (UGT2B7, UGT2B15, and UGT2B17) to efficiently catalyze *N*‐carbamoyl glucuronidation of lorcaserin, with UGT2B15 being the most efficient variant [138] (Scheme 8b). *N*‐Carbamoyl glucuronides have been reported for several amine‐based drugs, including sertraline [139], rimantadine [140], tocainide [141], AZD4831 [142] and sitagliptin [143] (Scheme 8b).

In many cases, UGT‐mediated *N‐*carbamoyl glucuronidation is stereoselective. For example, Bhattacharya et al. identified that *N*‐carbamoyl glucuronidation occurred enantioselectively toward the (*R*)‐enantiomer of AZD4831, a novel myeloperoxidase inhibitor [144]. Similarly, Khan et al. reported that UGT‐mediated *N*‐carbamoyl glucuronidation occurred exclusively with the (+)‐(*S)‐*enantiomer of primaquine [86]. UGT‐mediated *N*‐carbamoyl glucuronidation has inspired the development of new stereoselective synthetic routes for the *N*‐glycoconjugation of amine‐based drugs. This approach involves the coupling of amine‐derived carbamate anions with glycosyl halides and has been demonstrated across 100 amine‐containing drugs [145].

Flavin‐containing monooxygenases (FMOs) are frequently identified as major non‐CYP, or non‐UGT DMEs. Of the five human FMO isoforms characterized (FMO1‐5), FMO5 has been shown to exhibit atypical Baeyer–Villiger monooxygenase activity [146, 147]. FMO 5 catalyzes the Baeyer–Villiger oxidation (BVO) of the carbonyl moiety of the nabumetone ketol intermediate [148]. Recombinantly expressed FMO5 in *E. coli* has been exploited biocatalytically for the BVO of several aliphatic, cyclic, and aromatic carbonyl compounds, including nabumetone and pentoxifylline (Scheme 8c) [147, 149]. FMO5 was also shown to catalyze the sulfoxidation of thioanisole (Scheme 8c) [147]. More broadly, FMOs, including microbial FMOs, have recently been exploited for enantioselective sulfoxidation [150, 151].

Epoxide hydrolases catalyze the hydrolysis of epoxides to diols. The biomedical and biocatalytic potential of epoxide hydrolases has attracted increasing attention in recent years [152]; however, their substrate specificity remains poorly understood. The microsomal epoxide hydrolases (MEH, EPHX1) and soluble epoxide hydrolases (EPHX2) differ in expression patterns, enzymatic activity, and substrate scope. Epoxide hydrolase‐mediated ring opening of oxetanes to yield 1,3‐diols is a rare transformation but has recently gained attention. Li et al. identified the M1 metabolite (an oxetanyl ring‐opened diol analog) from spiro‐oxetane‐containing compound AZD1979 as a product of epoxide hydrolysis catalyzed by MEH [153]. Importantly, this represented the first reported example of a non‐epoxide substrate metabolized by MEH. This product was formed via hydration and ring‐opening of the oxetanyl moiety of AZD1979 (Scheme 8d). Hayes and colleagues further evaluated the human recombinant MEH against a panel of oxetane‐containing compounds, providing insights into the reactivity trends within this substrate class [154].

The oxetane ring has recently attracted significant interest as an emerging medicinal chemistry scaffold [155, 156], as it can be exploited to modulate drug metabolism pathways [157]. As such, biocatalytic routes enabling the synthesis of enantiopure oxetane‐based compounds, for example, through kinetic resolution using MEH‐type ring‐opening reactions, will improve access to enantiopure oxetanes. In this regard, and perhaps inspired by MEH‐catalyzed oxetane ring opening, Zhao et al. identified and engineered limone epoxide hydrolases (LEHs) for enantiocomplementary kinetic resolution of oxetanes [158]. The (*S*)‐selective CH55‐LEH and (*R*)‐selective *Re*‐LEH and their engineered variants were highly stereoselective and enabled enantiodivergent kinetic resolution of oxetanes, providing access to enantioenriched (*R*)‐ and (*S*)‐1, 3‐diols with up to 99% ee (Scheme 8d) [158].

AZD1979 was also shown to undergo a direct Phase II glutathione conjugation, without prior Phase I activation. Glutathione conjugate formation was catalyzed by recombinant human glutathione S‐transferases (GSTs), including GST A1, A2‐2, M1a, M2‐2, T1‐1, and a human placental GST, with GST A2‐2 displaying superior activity. NMR analysis confirmed the formation of the amino‐thioether conjugate regioisomer **M12** via GST‐mediated glutathione attack at the α‑carbon adjacent to the azetidine nitrogen, leading to the ring opening and formation of the amino‐thioether glutathione conjugate [37] (Scheme 8e).

Carbon‐carbon bond cleavage is a rare transformation for drug metabolizing CYPs; however, CYP1A2 and CYP3A4 have been reported to catalyze such reactions. For example, Varfaj et al. reported that CYP1A2 catalyzes C–C bond cleavage, converting nabumetone to its active metabolite, 6‑methoxy‑2‑naphthylacetic acid (6‑MNA). This pathway involves initial 3‐hydroxylation, followed by C–C cleavage to the corresponding aldehyde, and subsequent oxidation of the aldehyde to the carboxylic acid. These steps were shown to be catalyzed by CYP1A2 [148] (Scheme 8f). Li and coworkers reported a regioselective CYP3A4/5‐mediated C–C cleavage of pexidartinib. In this case, the C–C bond cleavage occurs through ipso‐addition rather than Baeyer–Villiger oxidation [159]. The CYP3A4/5‐mediated ipso‐oxygenation was reported to be highly regioselective, targeting sites that might be less accessible using conventional synthetic methods [159], Scheme 8e. Other CYPs, including those involved in sterol metabolism such as CYP51, CYP11A, CYP17A, CYP19A, and CYP125, have been shown to catalyze C–C bond cleavage [160, 161, 162]. Similarly, the bacterial CYP199A4 (F182A variant) has been engineered to catalyze regioselective hydroxylation of ketones and subsequent C–C bond cleavage of 4‐propionyl‐ and 4‐(2‐oxopropyl)‐benzoic acids to yield the corresponding aldehyde, with hydrogen peroxide acting as the oxidant [163].

### 8.3 Stereoselectivity Predictions: A Shared Interest Between DMPK and Biocatalysis Fields

As stereoselectivity investigations become increasingly integrated into broader drug metabolism studies and adjacent fields such as biocatalysis, the availability of high‐quality datasets is crucial for developing reliable AI models capable of predicting stereoselectivity across multiple enzyme classes. Transfer of learning between these adjacent fields in stereoselectivity prediction is therefore expected to accelerate progress in this area.

The extent and quality of data required to train robust algorithms for metabolite identification and stereoselectivity prediction necessitate systematic experimental screening campaigns extensive enough to generate such datasets. However, such studies are resource‐intensive and prohibitively expensive for most academic laboratories. A prototype of this approach was recently demonstrated by the AstraZeneca DMPK team, who generated and analyzed metabolite profiles from incubations of 120 compounds with cryopreserved, pooled primary human, dog, and rat hepatocytes [164]. In parallel, complementary in vivo studies were also conducted in dogs (beagles) and rats, with plasma and urine samples collected and their metabolite profiles determined [164]. The resulting datasets were subsequently used to develop an ML‐model for predicting sites of metabolism [165].

As an alternative to such resource‑intensive prospective studies, datasets from retrospective analyses of drug metabolism studies can also be valuable for ML training [165]. Over several decades, DMPK groups within major pharmaceutical companies have accumulated extensive, high‐value metabolite identification (MetID) data. These teams often maintain detailed repositories of MetID data [16], although such datasets are rarely accessible to the public. Making these data available, whether through industry–academic collaborations or public release, would provide a strong foundation for developing AI models for metabolite prediction, including for stereoselective biotransformation reactions. A recent collaboration between an AstraZeneca DMPK team and Kirchmair's group illustrates the potential of this approach. Their retrospective analysis of human hepatocyte assays enabled systematic mapping of sites of metabolism and associated functional groups or moieties across 217 compounds. A key challenge in using retrospective datasets for ML training is ensuring data consistency and compatibility, particularly when datasets originate from different sources. Chen et al. emphasized that differences in experimental and analytical methodologies, as well as variations in the chemical space represented, must be carefully considered and compatibility checks performed when using datasets from retrospective studies [51].

Stereoselectivity prediction is an area of shared research interest for both the DMPK and biocatalysis fields. Predictive tools capable of accurately forecasting the stereochemical outcomes of diverse enzyme classes, as well as stereoselective transformations mediated by subcellular fractions, would significantly advance both areas. Most enzymes display characteristic patterns of stereoselectivity and tend to retain these patterns across different substrates once the influence of Cahn–Ingold–Prelog priorities is accounted for. Systematic mapping of stereoselectivity across multiple enzymes within the same family, including orthologs from different species [166], using large sets of chiral or prochiral substrates and drug candidates would therefore provide high‐quality datasets for training stereoselectivity prediction models for any member of that enzyme family.

For DMEs affected by genetic polymorphisms, it is also important to map the effects of mutations on stereochemical outcomes. This mirrors the common practice in biocatalysis, where key mutations are routinely investigated to understand and optimize stereoselectivity. The importance of this approach is well established, since even a single‐point mutation can lead to a dramatic shift in stereochemical preference [167, 168, 169, 170, 171, 172]. The stereoselective properties of subcellular fractions should also be characterized and rationalized in relation to the enzyme expression profiles within those fractions and from the source organs or tissues. Datasets generated in adjacent fields for the same enzyme families, for example, stereoselectivity data for drug metabolizing human monoamine oxidases (MAOs) and those from fungal, bacterial, or mammalian homologues used in biocatalysis [173], can be combined after appropriate compatibility assessments. Such integrated datasets would provide a strong foundation for training algorithms capable of predicting stereoselective MAO‐catalyzed amine oxidation whether within drug metabolism or biocatalysis contexts.

## 9 Summary and Outlook

Stereoselectivity is an inherent property of drug metabolizing enzymes (DMEs), and data from both in vitro and in vivo studies show that enantiomers of numerous chiral drugs are metabolized at different rates. Our analysis of trends in stereoselective drug metabolism studies shows that the most frequently used in vitro approach for investigating stereoselectivity involves incubating racemates, as well as individual enantiomers of a chiral drug, with subcellular fractions and/or isolated recombinant enzymes. The evidence reviewed here demonstrates that the rate of (*R*)‐ versus (*S*)‐metabolism can vary depending on the type of subcellular fraction or tissue used for biotransformation, as the expression levels and localization of DMEs differ across tissues and organs. Similarly, orthologs of human DMEs in frequently employed animal models sometimes exhibit stereoselectivity opposite that observed in humans. Differences in stereoselectivity across tissues and cell types emphasize the importance of multi‐tissue and multi‐organ studies. Beyond enzyme expression, factors such as incubation conditions, interindividual variability, cell or tissue preparation methods, and physiological state of cells or tissues must be carefully controlled. Adoption of existing frameworks for in vitro metabolism study design and metabolite identification [18, 104, 174] could help reduce experimental variability and enhance translational relevance.

Systematic mapping of stereoselective metabolic properties across drug libraries and major DMEs, including relevant subcellular fractions from multiple metabolic sites and animal models, is also needed, as recently demonstrated by the AstraZeneca DMPK group [164]. Establishing in vitro stereoselectivity patterns that correlate with in vivo metabolism and clearance is essential to confirm the physiological relevance of in vitro stereoselectivity investigations. Standardized datasets generated from these studies could enable the development of reliable AI models capable of predicting stereoselective metabolism across enzymes, cell types, species, and genotypes.

Translation from animal models to humans remains difficult due to interspecies differences in stereoselective metabolism. Although CYP orthologs in animal models are reasonably well defined, equivalence or orthologs of other DMEs, including UGTs, remain less well established. Humanized animal models, such as liver‐humanized mice, represent a promising strategy to improve the predictive power of animal model‐based drug metabolism studies, and studies using these models should also incorporate stereoselectivity investigations.

Investigations into the effects of genetic polymorphisms on stereoselectivity of DMEs can improve outcomes in personalized medicine. However, many in vivo genetic studies remain ethnically skewed, limiting their generalizability. As genotype alone does not fully explain stereoselective outcomes, in vivo metabolism studies investigating the effect of polymorphism should be supported by mechanistic insights from in vitro approaches, and by investigating the impact of rationally designed point mutations on stereoselectivity [169, 170]. Similarly, mapping enzyme systems involved in chiral inversion and their enantioselectivities may further inform the design of compounds that are less susceptible to chiral inversion.

Drug‐drug interactions (DDIs) and stereoselective inhibition of DMEs by concomitantly administered drugs pose an additional challenge, as they can alter stereoselective metabolic profiles and lead to enantiomer‐specific accumulation with potential clinical consequences. The complexity of these interactions underscores the need for careful, detailed pharmacokinetic studies, as well as regulatory consideration and guidance.

Developing AI tools that can accurately predict stereoselectivity or stereochemical outcomes of biotransformation reactions remains a major unmet need and an area of shared interest for both DMPK and biocatalysis fields. Progress will require data sharing and the adoption of standardized methodologies for determining stereoselectivity so that compatible datasets can be generated and used across disciplines. Recent initiatives, such as the STRENDA Biocatalysis Guidelines [175] and the harmonized framework for in vitro metabolite identification proposed by Savaryn et al. [106], represent important steps toward protocol standardization; however, neither framework currently places emphasis on stereoselectivity data. Coordinating these efforts and establishing unified standards for reporting stereoselective biotransformations would support the creation of large, compatible datasets suitable for developing AI tools that can reliably predict stereoselectivity.

Finally, sustainable biosynthetic strategies are needed to access individual enantiomers of drug metabolites for pharmacological and toxicological evaluation as conventional chemical synthesis and chiral separation methods are often poorly stereoselective or time‐consuming, respectively. Stereoselective biotransformation provides a practical link between biocatalysis and DMPK. On the one hand, the advances in the development and engineering of novel biocatalytic routes [166, 171, 176, 177] can enable sustainable, stereoselective, and scalable metabolite synthesis of key chiral metabolites for DMPK studies. On the other hand, novel biotransformation reactions and the associated DMEs are frequently identified in DMPK studies; these transformations can inspire the design of novel stereoselective biocatalytic and biomimetic synthetic methods. The associated DMEs can also be engineered for use in both metabolite and API synthesis. A key bottleneck in exploiting human and animal DMEs for synthetic applications is whether they can be efficiently expressed in the *E. coli*, *Saccharomyces cerevisiae*, or *Pichia pastoris* expression systems commonly used for protein production in biocatalysis. Although several DMEs have been successfully expressed in *E. coli*, many enzyme families are still routinely produced in insect or mammalian cells. For specialized enzymes that catalyze rare and synthetically useful reactions, expression trials using different vectors (including those incorporating solubilizing tags) and optimized *E. coli* [178], yeast, or *Pichia* strains designed for human or animal protein expression may enable more sustainable and cost‐effective protein production for biocatalytic applications.

Together, these factors and the shared interests underscore the need for cross‐disciplinary collaboration between biocatalysis and DMPK scientists in both industry and academia.

## 10 Method for Systematic Literature Search

A comprehensive search was conducted amongst electronic databases, including Embase, Medline, PubMed, and Web of Science. To understand current trends in stereoselective drug metabolism (SDM), only articles published from January 2014 to February 2025 were retrieved. Concepts relating to the focus of this review were used to generate keyword combinations such as “chirality” OR “stereoselectivity” OR “enantioselectivity” AND “drug metabolism” OR “drug biotransformation” AND “chiral drug” OR “chiral pharmaceutic*” AND “pharmacology.” A complete list of search terms and their combinations used, organized by concept and electronic database, is provided in the Supporting Information (Table S1). Medical subject headings (MeSH terms) were “exploded” to retrieve results that were indexed under more specific related terms. Boolean operators, for example, AND/OR, enabled the combinations of concepts, while asterisks offered truncations that broadened the selection of results retrieved.

Before screening, the identified records were exported into Excel 2024, and duplicates were removed. Two reviewers (GAO and GAA) screened the literature, based on titles and abstracts, and studies not directly relevant to the subject of the Systematic Review were further excluded. The full texts of the remaining studies were retrieved and screened to further carefully examine eligibility. Any disagreements were resolved via discussion between the two reviewers.

The following inclusion criteria were used in the selection of studies to be included: (1) focused on the metabolism of a chiral or prochiral small‐molecule drug, (2) investigated metabolism through DMS, (3) reported significant SDM, (4) written in the English language, and (5) publication year no older than 2014. Studies were excluded from this review based on the following exclusion criteria: (1) not focused on a chiral or prochiral drug, (2) herbal/traditional medicine, (3) primary use of drug in non‐physiological settings, for example, the environment, (4) no significant stereoselective metabolism reported, (5) focus of the study was on enantiomer synthesis or separation methodology, (6) no pharmacokinetic application with presented metabolism data, (7) non‐primary research, and (8) non‐published and grey literature.

For data extraction, the selected studies were summarized and grouped by concept for thematic analysis. The concepts covered included chiral inversion, stereodivergent metabolic outcomes dependent on cell type, species, genetic polymorphisms, and enzyme inhibition/induction via DDIs. One reviewer (GAO) extracted the following data from the selected studies: first author, publication year, study design, (pro‐)chiral drug name and chemical structure, responsible DME, enzymatic metabolic pathway, metabolite name and chemical structure, and any significant observed stereoselectivity.

The literature search yielded 732 studies. In total, 568 duplicates were removed. Studies were screened out based on the relevance of their title (*n* = 60) or abstract (*n* = 26). A total of 78 primary research articles were assessed for eligibility via full‐text reading, with all texts being retrieved. Following full‐text assessment, 35 studies were excluded for meeting the exclusion criteria, including no significant metabolic stereoselectivity reported (*n* = 12), environmental primary use of the drug (*n* = 13), or the drug being classed as a traditional/herbal medicine (*n* = 6). Ultimately, 43 studies were selected for final inclusion in this systematic review. Details of the study selection process are outlined in the Preferred Reporting Items for Systematic Reviews and Meta‐Analyses (PRISMA) flow diagram [179] (Figure S1).

## Conflicts of Interest

The authors declare no conflicts of interest.

## Supporting information



The Supporting Information contains the PRISMA flow diagram detailing the literature selection process across electronic databases, the key search terms used for each database, and tables summarizing sites of metabolism with the corresponding literature references.
**Supporting File**: anie72311‐sup‐0001‐SuppMat.docx.

## Data Availability

Data sharing is not applicable to this article as no new data were created or analyzed in this study.
