# Supplementary material for: Stereoselective Biotransformation: Transfer of Learning to Advance Drug Metabolism and Biocatalysis
Source: Angew Chem Int Ed Engl. 2026 Apr 24;65(25):e26152. doi: 10.1002/anie.202526152 (PMC13266941; doi:10.1002/anie.202526152)
Supplement: Supplementary file 1 — The Supporting Information contains the PRISMA flow diagram detailing the literature selection process across electronic databases, the key search terms used for each database, and tables summarizing sites of metabolism with the corresponding literature references. Supporting File: anie72311‐sup‐0001‐SuppMat.docx. [file ANIE-65-e26152-s001.docx]

**Supporting Information**

**Stereoselective Biotransformation: Transfer of Learning to Advance Drug Metabolism and Biocatalysis**

Grace A. Okunlola, and Godwin A. Aleku*

Institute of Pharmaceutical Science, King’s College London, Franklin-Wilkins Building, 150 Stamford Street, London SE1 9NH, United Kingdom.
E-mail: godwin.aleku@kcl.ac.uk


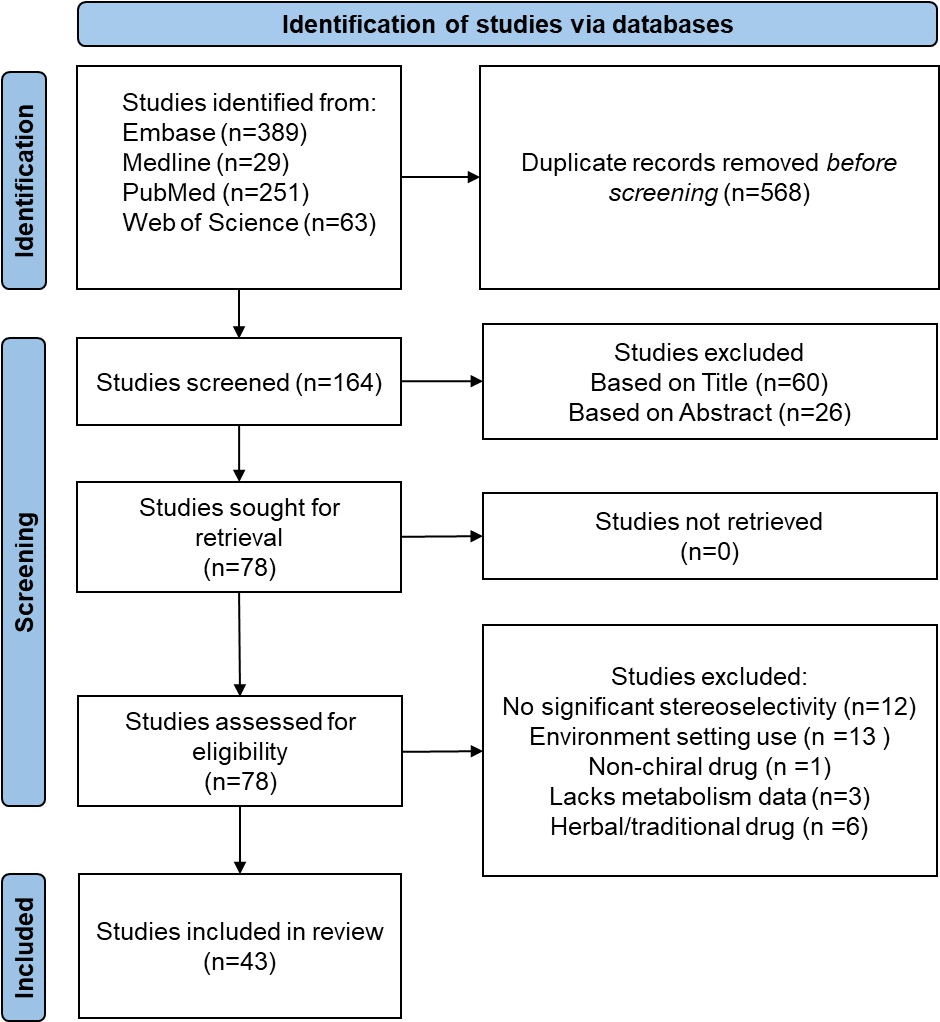


**Figure S1.** PRISMA flow diagram outlining the process of primary literature selection from electronic databases (n=4).

**Table S1.** Key search terms used in the literature search per electronic database

| Electronic  Database | Search Type | Concept | Keywords | Total Results |
| --- | --- | --- | --- | --- |
| **Embase** | Advanced | Stereoisomerism | exp Stereoisomerism/ OR exp Stereoselectivity/ OR Enantioselectivity/ OR exp Chirality/ OR exp Stereospecificity OR exp Racemic Mixture OR Stereoselective Discrimination.mp OR Enantioselective Discrimination.mp | 138,597 |
|  |  | Drug Metabolism | exp Drug Metabolism/ OR Drug Biotransformation.mp OR Stereoselective Metabolism.mp OR Stereoselective Biotransformation.mp OR Enantioselective Metabolism.mp OR Enantioselective Biotransformation.mp OR Product Stereoselectivity.mp OR Substrate Stereoselectivity.mp OR Product Enantioselectivity.mp OR Substrate Enantioselectivity.mp OR Enantiomeric Interaction.mp OR Chiral Inversion.mp | 208,511 |
|  |  | Chiral Pharmaceutics | Chiral Drug*.mp OR Chiral Pharmaceutic* OR Enantiomer* Drug*.mp OR Stereoisomer* Drug*.mp OR Prochiral.mp OR Enantiomer*.mp OR Stereoisomer*.mp | 106,757 |
|  |  | Pharmacology | exp Pharmacology/ | 5,475,204 |
|  |  | | | 389 |
| **MedLine** | Advanced | Stereoisomerism | Exp Stereoisomerism/ OR Stereoselectivity.mp OR Enantioselectivity.mp OR Chiral*.mp OR Stereospecific*.mp OR Racemate.mp OR Racemic Mixture.mp OR Stereoselective Discrimination.mp OR Enantioselective Discrimination.mp | 157302 |
|  |  | Drug Metabolism | Drug Metabolism.mp OR Drug Biotransformation.mp OR Stereoselective Metabolism.mp OR Stereoselective Biotransformation.mp OR Product Stereoselectivity.mp OR Substrate Stereoselectivity.mp OR Product Enantioselectivity.mp OR Substrate Enantioselectivity.mp OR Enantiomeric Interaction.mp OR Chiral Inversion.mp | 14,820 |
|  |  | Chiral Pharmaceutics | Exp Pharmaceutical Preparations/ AND Chiral Drug.mp OR Chiral Pharmaceutic*.mp OR Enantiomer* Drug.mp OR Stereoisomer* Drug.mp OR Prochiral.mp OR Enantiomer*.mp OR Stereoisomer*.mp | 118,308 |
|  |  | Pharmacology | exp Pharmacokinetics/ OR Pharmacodynamic.mp | 377,282 |
|  |  | | | 29 |
| **PubMed** | Basic | Stereoselective Metabolism AND Chiral Drug NOT review. | | 251 |
| **Web of Science** | Advanced | Stereoisomerism | Stereoselectivity OR Stereospecificity OR Enantioselectivity OR Enantiospecificity | 54,442 |
|  |  | Drug Metabolism | CYP450 OR Cytochrome 450 OR Drug Metabolism OR Drug Biotransformation OR Stereoselective Metabolism OR Enantioselective Metabolism OR Product Substrate Stereoselectivity OR Product Substrate Enantioselectivity OR Chiral Recognition OR Chiral Inversion | 300,354 |
|  |  | Chiral Pharmaceutics | Chiral* OR Prochiral* OR Chiral Drug OR Chiral Pharmaceutic* OR Chiral Xenobiotic OR Racemate OR Racemic Mixture OR Enantiomer* OR Stereoisomer* | 301,445 |
|  |  | Pharmacology | Pharmacology OR Pharmacokinetics OR Drug Disposition OR Pharmacodynamics | 415,043 |
|  |  | | | 63 |

*Note.* Exp = exploded term. / = Medical Subject Headings.

**Table S2**. Chiral drugs and subsequent metabolites demonstrating CYP-mediated phase I stereoselective biotransformation. Note: Chiral centres indicated by Asterix and metabolism sites with blue highlight. Abbreviations: CYP = Cytochrome P450. POR = Cytochrome P450 oxidoreductase.

| Drug Name and Chemical Structure | Metabolic Pathway | Responsible enzyme (Stereoselectivity) | Metabolite Name | Ref |
| --- | --- | --- | --- | --- |
|  | Dehydrogenation | CYP3A4*(S>R)*  CYP2C9*(R>S)*  CYP2C19*(R>S)* | *(R/S)-*Amlodipine Pyridine Derivate | ^[1]^ |
|  | *N*-Demethylation | CYP2D6 *(S>R)*  Canine CYP2D15 *(S>R)*  Canine CYP3A12 *(S>R)* | *(R/S)-*N-desmethyl-citalopram | ^[2]^ |
|  | *N*-Demethylation | CYP2D6 *(S>R)*  Canine CYP2D15 *(S>R)*  Canine CYP3A12 *(S>R)* | *(R/S)-*N-didesmethyl-citalopram | ^[2]^ |
|  | 1+2) Hydroxylation | CYP3A4*(S>R)*  CYP2C19*(R>S)*  CYP2C8*(R>S)* | 1) *(R,R/S,S)-*Doxazosin Metabolite M21  2) *(R,R/S,S)-*Doxazosin Metabolite M22 | ^[3]^ |
|  | Dehydration | CYP2C8*(S>R)* | Doxazosin Metabolite M18 | ^[3]^ |
|  | 8’-Hydroxylation | CYP2B6*(S>R)* | *(R/S)-*8-Hydroxyefavirenz | ^[4]^ |
|  | *N*-Demethylation | CYP2C8*(R>S)*  CYP2C9*(R>S)*  CYP2D6*(R>S)* | *(R/S)-*Norfluoxetine | ^[5]^ |
|  | *N*-Demethylation | Canine CYP3A12*(R>S)*  CYP2B6 (major)*(S>R)*  POR (minor)*(S>R)* | *(R/S)-*Norketamine | ^[6]^ |
|  | 1) *S*-Dealkylation | CYP3A4*(R>S)* | Lesinurad Thiol Metabolite M6 | ^[7]^ |
|  | 2+3) Hydroxylation | CYP2C9*(S>R)* | 2) Lesinurad Expoide Intermediate Metabolite  M3c  3) Lesinurad Metabolite M3 | ^[7]^ |
|  | Racemisation | CYP*(S>R)* | *(R)-*Lansoprazole | ^[8]^ |
|  | Reduction | Carbonyl reductase *(R,S>R,R)* | *(R,S,R)-/(R,S,S)-*Loxoprofen alcohol | ^[9]^ |
|  | Racemisation | 2-APCoAE*(S,R/R,R>R,S/S,S)* | *(S,S/R,S)-*Loxoprofen | ^25]^ |
|  | *N*-Dealkylation | CYP3A4 (major)*(S>R)*  CYP2C9 (minor)*(S>R)* | *(R/S)-*Desbutyllumefantrine | ^[10]^ |
|  | Racemisation | CYP*(S>R)* | *(R)-*Omeprazole | ^[8]^ |
|  | 1) *S*-Oxidation | CYP3A4*(S>R)* | *(R/S)-*Omeprazole Sulfone | ^[11]^ |
|  | 2) Hydroxylation | CYP2C19*(R>S)*  Marmoset CYP2C19*(S>R)* | 2.1) *(R/S)-*5-Hydroxyomeprazole  2.2) *(R/S)-*5’-O-Hydroxyomeprazole | ^[11]^ |
|  | Racemisation | CYP*(S>R)* | *(R)-*Pantoprazole | ^24]^ |
|  | Racemisation | CYP*(S>R)* | *(R)-*Rabeprazole | ^[8]^ |
|  | Unspecified Oxidation | CYP2B6*(S>R)* | *(R/S)-*Oxidised S002-333 Metabolite | ^[12]^ |
|  | *N*-Demethylation | CYP3A4/5*(R>S)*  CYP2C19*(R>S)* | *(R/S)-*N-mono-desmethylsibutramine | ^[13]^ |
|  | 1. *N*-Demethylation | CYP2B6*(S>R)*  CYP2C19*(R>S)* | *(R/S)-*N,N-didesmethylsibutramine | ^[13]^ |
|  | 1. Hydroxylation | CYP2B6*(S>R)*  CYP2C19*(S>R)* | *(R/S)-*N-mono-desmethylsibutramine hydroxyl M1 | ^[13]^ |
|  | 2-Keto reduction | CYP2C19*(Trans>Cis)* | Cis/Trans-Dihydro-Tetrabenazine | ^[14]^ |
|  | 1. 3’-Hydroxylation 2. 4’-Hydroxylation | CYP3A5*(S>R)* | 1) (3*R/S,*5*R/S)-*Tolvaptan-diol  2) (4*R/S,*5*R/S)-*Tolvaptan-diol | ^[15]^ |
|  | *O*-Demethylation | CYP2D6*(S,S>R,R)* | *(R/S)-*O-Desmethyl-tramadol | ^[16]^ |
|  | Hydroxylation | CYP2C8 *(Pro-R>Pro-S)*  Monkey CYP2C20 *(Pro-R>Pro-S)* | *(R/S)-*Gem-dimethyl Hydroxylated Tucatinib Metabolite M1 | ^[17]^ |
|  | *N*-Demethylation | CYP3A4*(S>R)* | *(R/S)-*Norverapamil | ^[18]^ |
|  | 1. 6’-Hydroxylation 2. 7’-Hydroxylation 3. 10’- Hydroxylation | CYP2C9*(S>R)* | *1) (R/S)-*6’-Hydroxywarfarin  *2+3) (R,R/S,S)-*7’/-10’-Hydroxywarfarin | ^[19]^ |

**Table S3.** Chiral drugs and subsequent metabolites demonstrating non-CYP-mediated phase I stereoselective biotransformation. Note: Chiral centres indicated by Asterix and metabolism sites with blue highlight. Abbreviations: 2APCoAE = 2-aryl propionyl-Coenzyme A epimerase. BChE = Butyrylcholinesterase. CES = Carboxylesterase. MEH = Microsomal Epoxide Hydrolase.

| Drug Name and Chemical Structure | Metabolic Pathway | Responsible enzyme (Stereoselectivity) | Metabolite Name | Ref |
| --- | --- | --- | --- | --- |
|  | Hydrolysis | BChE*(R>S)* | *(R/S)-*Bambuterol Monocarbamate | ^[20]^ |
|  | Hydrolysis | BChE*(R>S)* | *(R/S)-*Terbutaline | ^[20]^ |
|  | Hydrolysis | CES1*(R>S)* | Indomethacin 3-chloro-1-phenylpropanoic acid | ^[21]^ |
|  | Hydrolysis | CES1*(R>S)* | Indomethacin 1-Phenylethanoic acid | ^[21]^ |
|  | Hydrolysis | MEH *(S>R)* | Lesinurad Dihydrodiol Metabolite M4 | ^[7]^ |
|  | Reduction | Carbonyl reductase *(R,S>R,R)* | *(R,S,R)-/(R,S,S)-*Loxoprofen alcohol | ^[9]^ |
|  | Racemisation | 2-APCoAE*(S,R/R,R>R,S/S,S)* | *(S,S/R,S)-*Loxoprofen | ^[9]^ |

**Table S4.** Chiral drugs and subsequent metabolites demonstrating phase II stereoselective biotransformation. *Note*. Chiral centres indicated by Asterix and metabolism sites with blue highlight. Abbreviations: SULT = Sulfotransferase. UGT = Uridine diphosphate-glucuronosyltransferase.

| Drug Name and Chemical Structure | Metabolic Pathway | Responsible enzyme (Stereoselectivity) | Metabolite Name | Ref |
| --- | --- | --- | --- | --- |
|  | *O*-Glucuronidation | UGT1A9*(R>S)*  UGT2B7*(R>S)*  UGT2B15*(S>R)* | (*R/S)-*4-Ipomeanol glucuronide | ^[22]^ |
|  | *N*-Glucuronidation | UGT2B10*(Dex>Levo)* | •*Dex*medetomidine Glucuronide  •*Levo*medetomidine Glucuronide | ^[23]^ |
|  | *O*-Glucuronidation | UGT1A1*(S>R)*  UGT1A9*(S>R)* | *(R/S)-*Propranolol Glucuronide | ^[23]^ |
|  | *O*-Glucuronidation | UGT2B10*(R>S)*  UGT1A4*(R>S)* | *(R/S)-*RO5263397/6 Glucuronide | ^[23]^ |
|  | *O*-Sulphonation | SULT1A3*(R>S)* | *(R/S)-*Salbutamol Sulphate | ^[24]^ |
|  | *O*-Glucuronidation | UGT2B17*(R>S)* | 1) *(S)-*Testosterone Glucuronide  2) *(R)-*Epitestosterone Glucuronide | ^[23]^ |
|  | *O*-Glucuronidation | UGT2B15 *(S>R)*  UGT1A9 *(S>R)* | *(R/S)-*Vasicine Glucuronide | ^[25]^ |

**Table S5.** Chiral drugs and subsequent metabolites demonstrating both phase I and II stereoselective biotransformation. *Note*. Chiral centres indicated by Asterix and metabolism sites with blue highlight. Abbreviations: 11β-HSD = 11β-hydroxysteroid dehydrogenase. 2APCoAE = 2-aryl propionyl-Coenzyme A epimerase. ADH = Alcohol dehydrogenase. AKR = Aldo-keto reductases. ALDH = Aldehyde dehydrogenase. COMT = Catechol-O-methyltransferase. CYP = Cytochrome P450. MAO = Monoamine oxidase. SULT = Sulfotransferase. UGT = Uridine diphosphate-glucuronosyltransferase.

| Drug Name and Chemical Structure | Metabolic Pathway | Responsible enzyme (Stereoselectivity) | Metabolite Name | Ref |
| --- | --- | --- | --- | --- |
|  | 1. 4’-Hydroxylation | CYP2B6(S>R) | (R,R/S,S)-Hydroxybupropion | ^[26]^ |
|  | 1. Reduction | 11β-HSD1(S>R)  AKR (R>S) | 2.1) (R,R/S,S)-Threohydrobupropion  2.2) (S,R/R,S)-Erythrohydrobupropion | ^[26]^ |
|  | O-Glucuronidation | UGT2B7(R,R>S,S)  UGT2B4(R,R) | (R,R/S,S)-Hydroxybupropion Glucuronide | ^[27]^ |
|  | O-Glucuronidation | UGT1A9(R,R>S,S)  UGT1A4(R,R>S,S)  UGT2B7(R,S>S,R)  UGT2B4(R,S>S,R) | 1) (R,R/S,S)-Threohydrobupropion Glucuronide  2) (S,R/R,S)-Erythrohydrobupropion Glucuronide | ^[27]^ |
|  | Hydroxylation | CYP(S>R) | 1) (R/S)-HSG4112 M1a/M2a/M2b  2) (R,R/S,S)-HSG4112 M1c | ^[28]^ |
|  | O-Glucuronidation | UGT(S>R) | (R/S)-HSG4112 Glucuronide M4 | ^[28]^ |
|  | 2-Hydroxylation | CYP(S>R) | (R/S)-2-Hydroxy-Ibuprofen | ^[29]^ |
|  | Racemisation | 2-APCoAE (R>S) | (S)-Ibuprofen | ^[29]^ |
|  | O-Glucuronidation | UGT(S>R) | (R/S)-Ibuprofen Glucuronide | ^[29]^ |
|  | O-demethylation | CYP2D6(S>R) | (R/S)-3,4-DHMA | ^[30,31]^ |
|  | O-methylation | COMT(S>R) | (R/S)-HMMA | ^[30,31]^ |
|  | O-Sulphonation | SULT(S>R) | 1) (R/S)-DHMA 3-Sulfate  2) (R/S)-DHMA 4-Sulfate | ^[30,31]^ |
|  | O-Glucuronidation | UGT(S>R) | (R/S)-HMMA Glucuronide | ^[30,31]^ |
|  | O-Sulphonation | SULT(S>R) | (R/S)-HMMA Sulfate | ^[30,31]^ |
|  | Ring Oxidation | CYP3A4(S>R)  CYP2C9(S>R) | (R/S)-X-Hydroxy-Praziquantel | ^[32]^ |
|  | 4’-hydroxylation | CYP2C19(S>R)  CYP3A4(S>R)  CYP1A2(R>S) | (R/S)-4-Hydroxy-Praziquantel | ^[33]^ |
|  | 1) *C*-Glucuronide Conjugation | UGT(S>R) | (R/S)-Praziquantel Glucuronide | ^[32]^ |
|  | 1) Oxidative deamination | MAO-A(R>S) | (R/S)-Primaquine Aldehyde Intermediate | ^[34]^ |
|  | 2) Quinoline ring hydroxylation | CYP2D6(R>S) | (R/S)-Hydroxylated Primaquine Metabolite | ^[34]^ |
|  | 3) N-Carbamoyl Glucuronidation | UGT(R>S) | (R/S)-Primaquine Carbamoyl Glucuronide | ^[34]^ |
|  | Aldehyde Dehydrogenation | ALDH(R>S) | (R/S)-Carboxyprimaquine | ^[35]^ |
|  | 1+2) Hydroxylation | CYP2C19(Pro-R>Pro-S)  CYP3A4(Pro-S>Pro-R)  CYP3A5(Pro-S>Pro-R) | 1) (R,R,R/R,R,S)-Tivantinib Metabolite M4/M5/M8 Precursor  2) (R,R,R/R,R,S)-Tivantinib Metabolite M8 Precursor | ^[36]^ |
|  | Alcohol dehydrogenation | ADH4(R,R,R>R,R,S) | (R,R)-Tivantinib Metabolite M6 | ^[36]^ |
|  | O-Glucuronidation | UGT1A9(R,R,S>R,R,R) | (R,R)-Tivantinib Metabolite M15/16 | ^[36]^ |
|  | Alcohol dehydrogenation | ADH4(R,R,R>R,R,S) | (R,R)-Tivantinib Metabolite M8 | ^[36]^ |

References.

[1] K. Krasulova, O. Holas, P. Anzenbacher, “Influence of Amlodipine Enantiomers on Human Microsomal Cytochromes P450: Stereoselective Time-Dependent Inhibition of CYP3A Enzyme Activity” *Molecules* **2017**, *22*, 1879.

[2] B. Rochat, E. Paus, C. Maitre, P. Baumann, “Citalopram in vitro metabolism in a beagle dog: A role for CYP2D15 in the production of toxic didesmethylcitalopram?” *Veterinární Medicína* **2023**, *68*, 135–144.

[3] D. Kong, Y. Tian, K. Duan, W. Guo, Q. Zhang, P. Zhang, Z. Yang, X. Qin, L. Ren, W. Zhang, “Elucidating a Complicated Enantioselective Metabolic Profile: A Study From Rats to Humans Using Optically Pure Doxazosin” *Front. Pharmacol.* **2022**, *13*, DOI 10.3389/fphar.2022.834897.

[4] P.-F. Wang, A. Neiner, E. D. Kharasch, “Efavirenz Metabolism: Influence of Polymorphic CYP2B6 Variants and Stereochemistry” *Drug Metab. Dispos.* **2019**, *47*, 1195–1205.

[5] Z. Wang, S. Wang, M. Huang, H. Hu, L. Yu, S. Zeng, “Characterizing the effect of cytochrome P450 (CYP) 2C8, CYP2C9, and CYP2D6 genetic polymorphisms on stereoselective N-demethylation of fluoxetine” *Chirality* **2014**, *26*, 166–173.

[6] F. A. Sandbaumhüter, R. Theurillat, W. Thormann, “Effects of medetomidine and its active enantiomer dexmedetomidine on N-demethylation of ketamine in canines determined in vitro using enantioselective capillary electrophoresis” *Electrophoresis* **2015**, *36*, 2703–2712.

[7] C. Yang, D. Zhou, Z. Shen, D. M. Wilson, M. Renner, J. N. Miner, J.-L. Girardet, C. A. Lee, “Characterization of Stereoselective Metabolism, Inhibitory Effect on Uric Acid Uptake Transporters, and Pharmacokinetics of Lesinurad Atropisomers” *Drug Metab. Dispos.* **2019**, *47*, 104–113.

[8] C. Tang, Z. Chen, X. Dai, W. Zhu, D. Zhong, X. Chen, “Mechanism of Reductive Metabolism and Chiral Inversion of Proton Pump Inhibitors” *Drug Metab. Dispos. Biol. Fate Chem.* **2019**, *47*, 657–664.

[9] S. Cao, X. Shi, S. Han, Y. Fu, X. Liu, P. Zhao, Z. Wang, “Study of the stereospecificity in the biotransformation of the four isomers of loxoprofen sodium in rats by chiral HPLC” *J. Sep. Sci.* **2024**, *47*, e2300562.

[10] B. B. Gabani, A. Dixit, V. Kiran, R. M. Bestha, B. Narayanan, N. R. Srinivas, Ramesh, Mullangi, “Enantioselective in vitro ADME, absolute oral bioavailability, and pharmacokinetics of (−)-lumefantrine and (+)-lumefantrine in mice” *Xenobiotica* **2021**, *51*, 202–209.

[11] S. Xia, H. Hirao, “Stereo- and Regioselective Metabolism of the Omeprazole Enantiomers by CYP2C19: Insights from Binding Free Energy and QM/MM Calculations in a Curtin–Hammett Framework” *ACS Catal.* **2025**, *15*, 3284–3294.

[12] M. Bhateria, R. Ramakrishna, S. K. Puttrevu, A. K. Saxena, R. S. Bhatta, “Enantioselective inhibition of Cytochrome P450-mediated drug metabolism by a novel antithrombotic agent, S002-333: Major effect on CYP2B6” *Chem. Biol. Interact.* **2016**, *256*, 257–265.

[13] D. D. Shinde, M.-J. Kim, E.-S. Jeong, Y.-W. Kim, J.-W. Lee, J.-G. Shin, Dong-Hyun, Kim, “Enantioselective N-Demethylation and Hydroxylation of Sibutramine in Human Liver Microsomes and Recombinant Cytochrome P-450 Isoforms” *J. Toxicol. Environ. Health A* **2014**, *77*, 1419–1430.

[14] M. Z. Bocato, F. de L. Moreira, N. C. P. de Albuquerque, C. M. de Gaitani, A. R. M. de Oliveira, “In vitro enantioselective human liver microsomal metabolism and prediction of in vivo pharmacokinetic parameters of tetrabenazine by DLLME-CE” *J. Pharm. Biomed. Anal.* **2016**, *128*, 528–537.

[15] S. Akutsu, Y. Mino, T. Naito, K. Hoshikawa, M. Saotome, Y. Maekawa, J. Kawakami, “Stereoselective interaction of tolvaptan with amiodarone under racemic metabolic impact by CYP3A5 genotypes in heart failure patients” *Eur. J. Clin. Pharmacol.* **2022**, *78*, 1311–1320.

[16] K. Suzuki, T. Naito, H. Tanaka, Y. Yamada, K. Itoh, J. Kawakami, “A Reversed-Phase Mode LC-MS/MS Method Using a Polysaccharide Chiral Selector for Simultaneous Quantitation of Each Enantiomer of Tramadol and its Metabolites in Human Plasma and Evaluation of CYP-Mediated Stereoselective Demethylation” *Ther. Drug Monit.* **2020**, *42*, 503–511.

[17] H. Sun, K. A. Cardinal, L. Wienkers, A. Chin, V. Kumar, C. Neace, C. Henderson, C. J. Endres, A. Topletz-Erickson, K. Regal, A. Vo, S. C. Alley, A. J. Lee, “Elimination of tucatinib, a small molecule kinase inhibitor of HER2, is primarily governed by CYP2C8 enantioselective oxidation of gem-dimethyl” *Cancer Chemother. Pharmacol.* **2022**, *89*, 737–750.

[18] N. Hanke, D. Türk, D. Selzer, S. Wiebe, É. Fernandez, P. Stopfer, V. Nock, T. Lehr, “A Mechanistic, Enantioselective, Physiologically Based Pharmacokinetic Model of Verapamil and Norverapamil, Built and Evaluated for Drug-Drug Interaction Studies” *Pharmaceutics* **2020**, *12*, 556.

[19] D. R. Flora, A. E. Rettie, R. C. Brundage, T. S. Tracy, “CYP2C9 Genotype-Dependent Warfarin Pharmacokinetics: Impact of CYP2C9 Genotype on R- and S-Warfarin and Their Oxidative Metabolites” *J. Clin. Pharmacol.* **2017**, *57*, 382–393.

[20] M. Pistolozzi, H. Du, H. Wei, W. Tan, “Stereoselective Inhibition of Human Butyrylcholinesterase by the Enantiomers of Bambuterol and Their Intermediates” *Drug Metab. Dispos.* **2015**, *43*, 344–352.

[21] M. Takahashi, D. Takani, M. Haba, M. Hosokawa, “Investigation of the chiral recognition ability of human carboxylesterase 1 using indomethacin esters” *Chirality* **2020**, *32*, 73–80.

[22] A. M. Teitelbaum, M. G. McDonald, J. P. Kowalski, O. T. Parkinson, M. Scian, D. Whittington, K. Roellecke, H. Hanenberg, C. Wiek, A. E. Rettie, “Influence of Stereochemistry on the Bioactivation and Glucuronidation of 4-Ipomeanol” *J. Pharmacol. Exp. Ther.* **2019**, *368*, 308–316.

[23] N. Milani, N. Qiu, S. Fowler, “Contribution of UGT Enzymes to Human Drug Metabolism Stereoselectivity: A Case Study of Medetomidine, RO5263397, Propranolol, and Testosterone” *Drug Metab. Dispos.* **2023**, *51*, 306–317.

[24] L. C. Harps, A. L. Jendretzki, C. A. Wolf, U. Girreser, G. Wolber, M. K. Parr, “Development of an HPLC-MS/MS Method for Chiral Separation and Quantitation of (R)- and (S)-Salbutamol and Their Sulfoconjugated Metabolites in Urine to Investigate Stereoselective Sulfonation” *Molecules* **2023**, *28*, 7206.

[25] Y. Zhu, W. Liu, S. Qi, H. Wang, Y. Wang, G. Deng, Y. Zhang, S. Li, C. Ma, Y. Wang, X. Cheng, C. Wang, “Stereoselective glucuronidation metabolism, pharmacokinetics, anti-amnesic pharmacodynamics, and toxic properties of vasicine enantiomers in vitro and in vivo” *Eur. J. Pharm. Sci.* **2018**, *123*, 459–474.

[26] N. O. Bamfo, J. B. Lu, Z. Desta, “Stereoselective Metabolism of Bupropion to Active Metabolites in Cellular Fractions of Human Liver and Intestine” *Drug Metab. Dispos.* **2023**, *51*, 54–66.

[27] B. T. Gufford, J. B. L. Lu, I. F. Metzger, D. R. Jones, Z. Desta, “Stereoselective Glucuronidation of Bupropion Metabolites In Vitro and In Vivo” *Drug Metab. Dispos.* **2016**, *44*, 544–553.

[28] I. Y. Bae, M. S. Choi, Y. S. Ji, S.-K. Yoo, K. Kim, H. H. Yoo, “Species Differences in Stereoselective Pharmacokinetics of HSG4112, A New Anti-Obesity Agent” *Pharmaceutics* **2020**, *12*, 127.

[29] H. Ikuta, A. Kawase, M. Iwaki, “Stereoselective Pharmacokinetics and Chiral Inversion of Ibuprofen in Adjuvant-induced Arthritic Rats” *Drug Metab. Dispos.* **2017**, *45*, 316–324.

[30] A. E. Steuer, C. Schmidhauser, E. H. Tingelhoff, Y. Schmid, A. Rickli, T. Kraemer, M. E. Liechti, “Impact of Cytochrome P450 2D6 Function on the Chiral Blood Plasma Pharmacokinetics of 3,4-Methylenedioxymethamphetamine (MDMA) and Its Phase I and II Metabolites in Humans” *PloS One* **2016**, *11*, e0150955.

[31] A. E. Steuer, C. Schmidhauser, E. H. Tingelhoff, Y. Schmid, A. Rickli, T. Kraemer, M. E. Liechti, “Impact of Cytochrome P450 2D6 Function on the Chiral Blood Plasma Pharmacokinetics of 3,4-Methylenedioxymethamphetamine (MDMA) and Its Phase I and II Metabolites in Humans” *PloS One* **2016**, *11*, e0150955.

[32] H. Wang, Z.-Z. Fang, Y. Zheng, K. Zhou, C. Hu, K. W. Krausz, D. Sun, J. R. Idle, F. J. Gonzalez, “Metabolic profiling of praziquantel enantiomers” *Biochem. Pharmacol.* **2014**, *90*, 166–178.

[33] N. N. Kapungu, X. Li, C. Nhachi, C. Masimirembwa, R. S. Thelingwani, “In vitro and in vivo human metabolism and pharmacokinetics of S‐ and R‐praziquantel” *Pharmacol. Res. Perspect.* **2020**, *8*, e00618.

[34] W. Khan, Y.-H. Wang, N. D. Chaurasiya, N. D. Nanayakkara, H. B. Herath, K. A. Harrison, G. Dale, D. A. Stanford, E. P. Dahl, J. D. McChesney, W. Gul, M. A. ElSohly, S. I. Khan, P. S. Fasinu, I. A. Khan, B. L. Tekwani, L. A. Walker, “Comparative single dose pharmacokinetics and metabolism of racemic primaquine and its enantiomers in human volunteers” *Drug Metab. Pharmacokinet.* **2022**, *45*, 100463.

[35] P. S. Fasinu, B. Avula, B. L. Tekwani, N. P. D. Nanayakkara, Y.-H. Wang, H. M. T. B. Herath, J. D. McChesney, G. A. Reichard, S. R. Marcsisin, M. A. Elsohly, S. I. Khan, I. A. Khan, L. A. Walker, “Differential kinetic profiles and metabolism of primaquine enantiomers by human hepatocytes” *Malar. J.* **2016**, *15*, 224.

[36] Y. Nishiya, D. Nakai, Y. Urasaki, H. Takakusa, S. Ohsuki, Y. Iwano, T. Yasukochi, T. Takayama, S. Bazyo, C. Oza, A. Kurihara, R. E. Savage, Takashi, Izumi, “Stereoselective hydroxylation by CYP2C19 and oxidation by ADH4 in the in vitro metabolism of tivantinib” *Xenobiotica* **2016**, *46*, 967–976.
